# Perception and prediction of the putting distance of robot putting movements under different visual/viewing conditions

**DOI:** 10.1371/journal.pone.0249518

**Published:** 2021-04-23

**Authors:** Gerrit Kollegger, Josef Wiemeyer, Marco Ewerton, Jan Peters

**Affiliations:** 1 Department of Human Sciences, Institute for Sport Science, Technische Universität Darmstadt, Darmstadt, Germany; 2 Intelligent Autonomous Systems Group, Department of Computer Science, Technische Universität Darmstadt, Darmstadt, Germany; University of Verona, ITALY

## Abstract

The purpose of this paper is to examine, whether and under which conditions humans are able to predict the putting distance of a robotic device. Based on the “flash-lag effect” (FLE) it was expected that the prediction errors increase with increasing putting velocity. Furthermore, we hypothesized that the predictions are more accurate and more confident if human observers operate under full vision (F-RCHB) compared to either temporal occlusion (I-RCHB) or spatial occlusion (invisible ball, F-RHC, or club, F-B). In two experiments, 48 video sequences of putt movements performed by a BioRob robot arm were presented to thirty-nine students (age: 24.49±3.20 years). In the experiments, video sequences included six putting distances (1.5, 2.0, 2.5, 3.0, 3.5, and 4.0 m; experiment 1) under full versus incomplete vision (F-RCHB versus I-RCHB) and three putting distances (2. 0, 3.0, and 4.0 m; experiment 2) under the four visual conditions (F-RCHB, I-RCHB, F-RCH, and F-B). After the presentation of each video sequence, the participants estimated the putting distance on a scale from 0 to 6 m and provided their confidence of prediction on a 5-point scale. Both experiments show comparable results for the respective dependent variables (error and confidence measures). The participants consistently overestimated the putting distance under the full vision conditions; however, the experiments did not show a pattern that was consistent with the FLE. Under the temporal occlusion condition, a prediction was not possible; rather a random estimation pattern was found around the centre of the prediction scale (3 m). Spatial occlusion did not affect errors and confidence of prediction. The experiments indicate that temporal constraints seem to be more critical than spatial constraints. The FLE may not apply to distance prediction compared to location estimation.

## Introduction

Human-robot interaction is gaining importance due to the increasing penetration of many areas by robotic devices, e.g., in rehabilitation, industrial production and motor skill learning in sports. The overlap of operating spaces between robots and humans is constantly growing. In this regard, an important question is how robots and humans can cooperate in an effective way. In an ideal cooperative scenario the perceptions and actions of humans and robots are perfectly coordinated taking the best of both, i.e., accuracy and precision of robot movements and flexibility and adaptability of human perception and action. This human-robot coordination has to be learned on both sides. Therefore, this paper focuses on motor skill learning of robot-human dyads. The learning of humans and robots raises numerous questions. There is an extensive research in human-robot interaction. Important current topics are—among others—the application of Artificial Intelligence and Machine learning (e.g., [1]), the significance of expert support (e.g., [2]), the impact of different forms of physical guidance (e.g., [3]) or the significance of motor variability (e.g., [4]). For example, if and under which conditions can robots profit from humans and vice versa. One possible scenario may be that at the beginning of the learning process humans teach robots by showing them a first solution and correcting big errors. After this initial phase of acquiring a rough solution (movement topology [5]) the robot may self-optimize its motions and may come up with solutions that are of value for the human. Taking the example of putting motion, the human guides the robot’s first attempts to hit the ball and approach the hole. The human teacher watches and corrects the motions if necessary. As soon as the robot motion has gained sufficient structural stability, a phase of self-optimization will follow. Finally, the robot presents its motions to the human who may use this information as visual or haptic guidance to improve her or his own putting (model or parameter learning [5]). In this hypothetic scenario, watching the robot motions plays an important role for the human to identify the relevant information in order to guide his or her own motion. A prototypical example of a motor learning application involving human-robot interaction in rehabilitation could start with a first demonstration of the movement to the robot, either by physical guidance of the robot or by visual demonstration to the robot. After this demonstration, the robot tries to imitate and further improve the movement. At specific time points, the robot presents the improved movements to the human, again either as visual demonstration or physical guidance, and the human may try to imitate the optimized robot movements. This human imitation may be measured and estimated by the robotic system which again adapts its movements to establish an individualized movement for the patient. This mutual support may lead to a more dynamic and personalized learning of both robot and human movements. Thus, sensorimotor rehabilitation becomes more flexible, more individual and more dynamic. Therefore, the question arises to what extent humans are able to perceive and predict robot motions.

In this paper, the issue of human perception of robot movements is addressed by two experiments where humans perceive and predict video-taped putting motions performed by a robot. Humans are able to perceive motions in several ways by monocular or binocular vision from dynamic and static input [6, 7]. One perceptual strategy is to keep the eyes still and let the moving object pass (afferent motion perception [8]). In this case, the brain constructs motions by integrating the successive projection of light on different retinal locations. This option is appropriate for the detection of motion, particularly in the periphery of the retina. However, due to the low visual acuity outside the fovea centralis, this strategy does not allow for identifying details of the moving object. As a consequence, velocity of a single moving object tends to be overestimated [8]. Respective experiments in psychophysics show, that the ability to discriminate velocity differences follows a U-shaped function favoring velocities of 8 to 64 degrees per second with a sensitivity threshold of about 5% for velocities over 2 degrees per second [9].

Another option allowing for more visual acuity and more accurate perception is to track the object with the eyes (efferent motion perception). In this case, the retinal location of the moving object is more or less stable and the impression of motion is elicited by integrating foveal and peripheral vision and proprioceptive feedback as well as control signals to the eye muscles. However, this second type of motion perception is limited to low object velocities, as object velocities beyond 70 degrees per second require saccadic eye movements [8].

Numerous experiments confirm that humans are able to perceive and classify biological movement, even if only few stimuli are available (“structure-from-motion studies” [10]). Furthermore, the human system for visual perception of motion seems to be hierarchically organized [11, 12]. In the point-light approach (review: [7]) introduced by Johansson [13], for example, human observers are able to judge the gender and identity of acting persons [14, 15], the weight of lifted boxes [16–18], specific motor actions like gait [19] and even emotions, intentions and attempted deceptions [18]. Based on these results, Runeson et al. [20] proposed the principle of “Kinematics Specify Dynamics” (KSD principle). The KSD principle claims that “movements specify the causal factors of events” [20]. However, human ability to perceive motions seems to be limited to nominal and ordinal judgement, i.e., classification and ranking. In a preliminary study, Ballreich [21] found that 29 expert coaches in jumping were able to rank kinematics (i.e., duration and velocity) and kinetics (i.e., force) but not joint angles of three successive high jumps. Cañal-Bruland and Williams [22] report evidence that distal cues, e.g. motion of the racquet, play an important role in predicting the directions of tennis strokes. The perception of human motion is subject to numerous influencing factors comprising features of the moving object (e.g., velocity, trajectory, size, and distance) and the observer (e.g., perceptual-motor experience, knowledge, and object-observer relation). An open question is whether the results found for biological motion transfer to non-biological motion. Recent evidence, both psychological and neurophysiological, suggests an interaction of distinct pathways processing biological and non-biological motion [23, 24]. Interestingly, the perception of animate motion can be elicited by simple visual stimuli with specific changes in velocity and direction [25]. Whereas the proposed experimental evidence confirms the hypothesis that kinematic parameters are integrated in the visual system to elicit nominal and ordinal judgements, it is unclear how well humans can estimate kinematics on a metric scale. The quantitative judgement of movements appears to be subject to numerous sources of error [8, 26]. For example, the “flash-lag effect” (FLE) indicates that the visual system commits errors when predicting the future position of moving objects [27, 28]. The FLE shows that under certain conditions humans tend to systematically overestimate the future position of moving objects. In soccer, for example, this perceptual error leads to a large number of false flags (offside [29]). The FLE is subject to numerous influences (review: [28]), for example, velocity of visual target, eye movements [30], attention [31] level of expertise, learning, and training [32], motion type, motion context [30], and expected dynamics [26]. Studies in neurosciences confirm an important contribution of area MT+ (medium temporal cortex) to the FLE [33], an area which is also involved in the perception of motion in general [34] and especially regarding motion direction [11, 35]. There are numerous theories trying to explain the FLE (for a review, see [28]). The purpose of this paper is to examine, whether and under which conditions humans are able to predict the putting distance of the putting motions of a robotic device. Due to the FLE it is expected that prediction errors increase with increasing putting velocity which is necessary to achieve different putting distances (hypothesis 1). Furthermore, the influence of watching different proportions of motion (temporal occlusion paradigm; [36]) as well as different vision conditions (spatial occlusion paradigm) is tested. It is expected that predictions are more accurate, more confident and faster if the human observers watch the full vision condition compared to the incomplete vision condition (temporal occlusion; hypothesis 2). In addition, it is expected that predictions are more accurate, more confident and faster with full vision (i.e. robot, club, and ball) compared to restricted vision after the impact, i.e., invisible ball or club (partial spatial occlusion; hypothesis 3) [36].

In the following, two consecutive experiments studies are described. In the first experiment, different movement sequences were presented at six different distances. Based on the results of the first experiment, the second experiment aimed at replicating the results of the first experiment and additionally manipulating the amount of information (visibility of club and ball in full vision condition).

## Experiment 1—Full vs. incomplete vision condition

In this experiment, hypothesis 1 and 2 are addressed by applying the temporal occlusion paradigm. Video sequences of robot putts at six different distances are presented to the participants. For each distance, videos are shown under two different visual conditions, i.e. full vision condition with visible robot, club, club head, and ball (F-RCHB; Note: F = full vision condition; I = incomplete vision condition; R = visible Robot; C = visible club; H = visible club head; B = visible ball), including preliminary, backswing, downswing, and follow-through phase, and incomplete vision condition with visible robot, club, club head, and ball (I-RCHB) including preliminary, backswing, and downswing phase until the club-ball impact (Note: The abbreviations are introduced here to make it easier to understand the abbreviations for the second experiment.).

### Materials and methods

#### Participants

Twenty healthy students (13 males and 7 females), aged 20 to 31 years, volunteered to participate in the experiment. Inclusion criteria was no previous experience with perceptual studies. Demographic data are presented in Table 1. This sample size was chosen because no reference study was available which allowed for calculating optimal sample size.

**Table 1:** Demographic data of the participants (mean±SD).

	n	Age [yr]	Height [cm]	Body mass [kg]	Handedness [left | right]
Female	7	24.5±3.5	169.0±4.9	61.4±3.6	1 | 6
Male	13	24.4±2.4	180.1±4.4	77.2±8.0	1 | 12
Total	20	24.5±2.7	176.2±7.2	71.7±10.2	2 | 18

All participants provided their experience (years of exercising and volume in hours per week) in four different groups of activities:

1. golf, field hockey, and similar;
2. returning games, e.g. tennis and volleyball;
3. ball games, e.g. soccer and basketball;
4. computer games.

Table 2 shows the information provided by participants regarding their previous experience.

**Table 2:** Experience in selective sports and computer games (mean±SD).

	Golf, field hockey and similar			Returning games			Ball games			Computer games		
	n	years	h/wk	n	years	h/wk	n	years	h/wk	n	years	h/wk
Female	0	–	–	2	13.5±12.0	7.7±10.2	2	8.5±2.1	7.0±4.2	2	3.2±2.0	0.5±0.5
Male	8^a^	2.1±1.7	2.2±2.6	9	5.2±7.5	2.1±1.5	12	13.6±8.1	4.1±2.5	11	9.0±5.1	4.0±3.7
Total	8	2.1±1.7	2.2±2.6	11	6.7±8.4	3.1±4.2	14	12.8±7.4	4.2±2.9	13	8.5±4.7	2.4±3.3

Means±SD were only calculated for participants reporting experience.

^a^ Experience in Golf, field hockey and similar sports was reported by a total of 10 participants, duration and volume were only reportet by 8 participants.

This experiment was conducted in accordance with the declaration of Helsinki in its latest version. All participants provided written informed consent before participation. The Ethical Committee of Technische Universität Darmstadt (TU Darmstadt) specifically approved this experiment.

#### Apparatus and task

**Fig 1:**
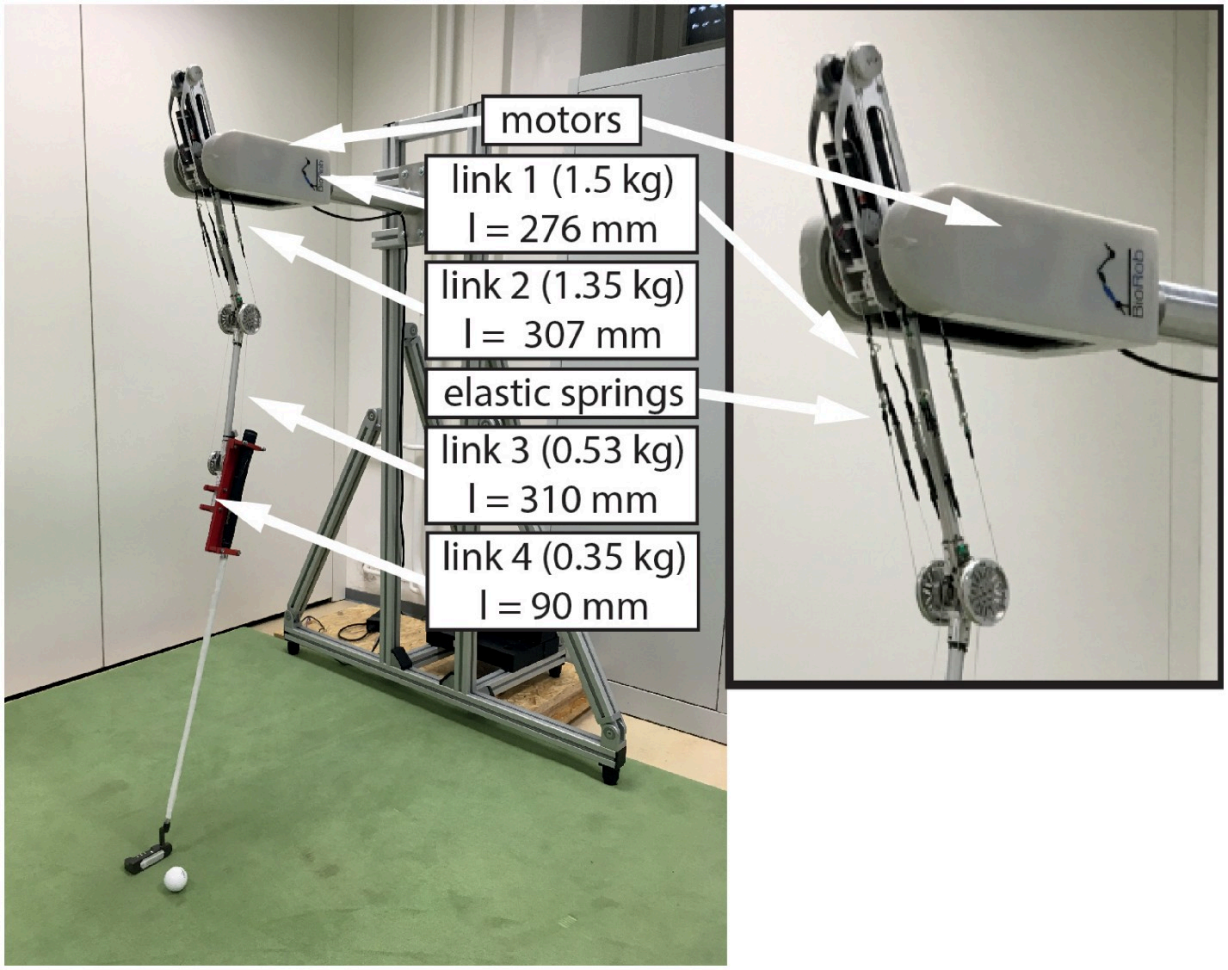
BioRob-system. BioRob with 4 DOF on a movable lightweight frame construction for experiments.

*BioRob system*. The BioRob robot arm was used as a technical platform for the experiments (see Fig 1). This system has four elastically actuated joints. Each joint is connected via four elastic springs with a separate actuator for each joint. The BioRob system was developed specifically for the physical interaction with humans. Due to its lightweight construction, the system generates low kinetic energy. The system is safe to use without collision detection. In order to adapt the system to the anthropometric properties of participants, the BioRob arm was attached to a special lightweight frame. This allows easy adjustment of the height and orientation of the robot arm [37].

**Fig 2:**
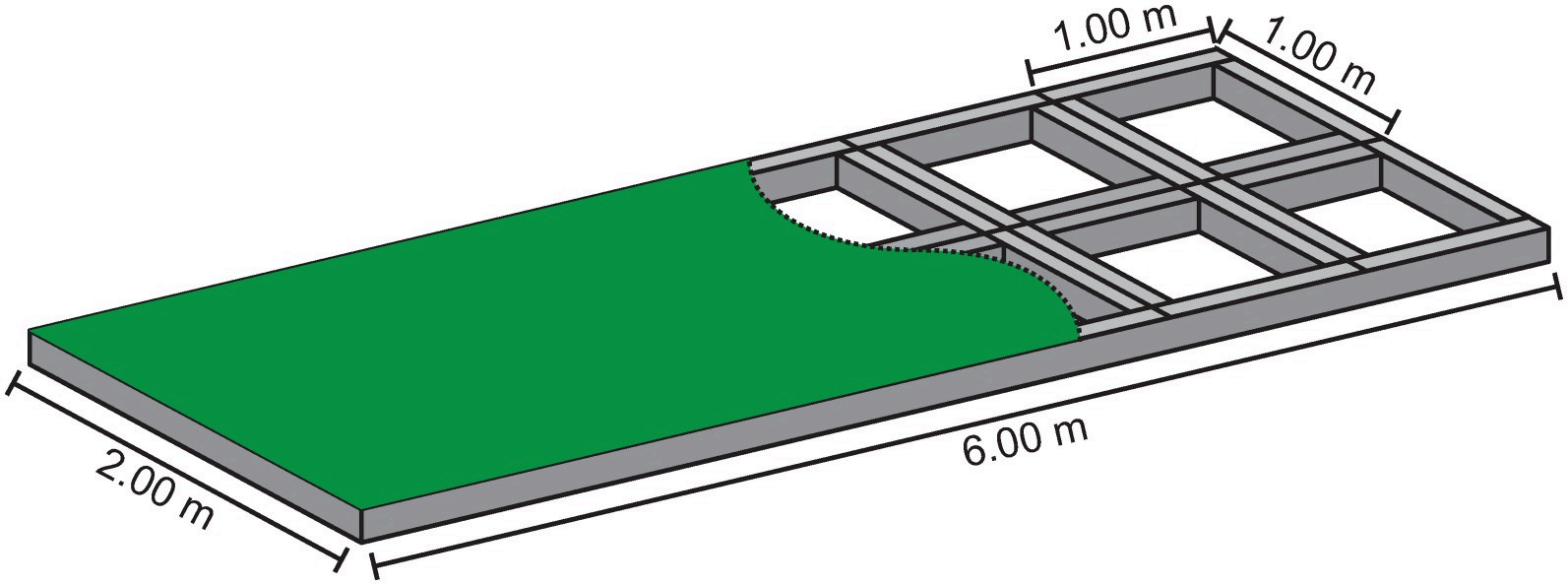
Artificial putting green. Schematic representation of the artificial putting green with substructure of aluminum profiles.

**Artificial putting green**. In order to enable a reproducible robot putt and a uniform rolling behavior of the golf balls, an artificial putting green was constructed (see Fig 2). The platform is six meters long and two meters wide. The surface consists of a short-pile carpet [37].

**Fig 3:**
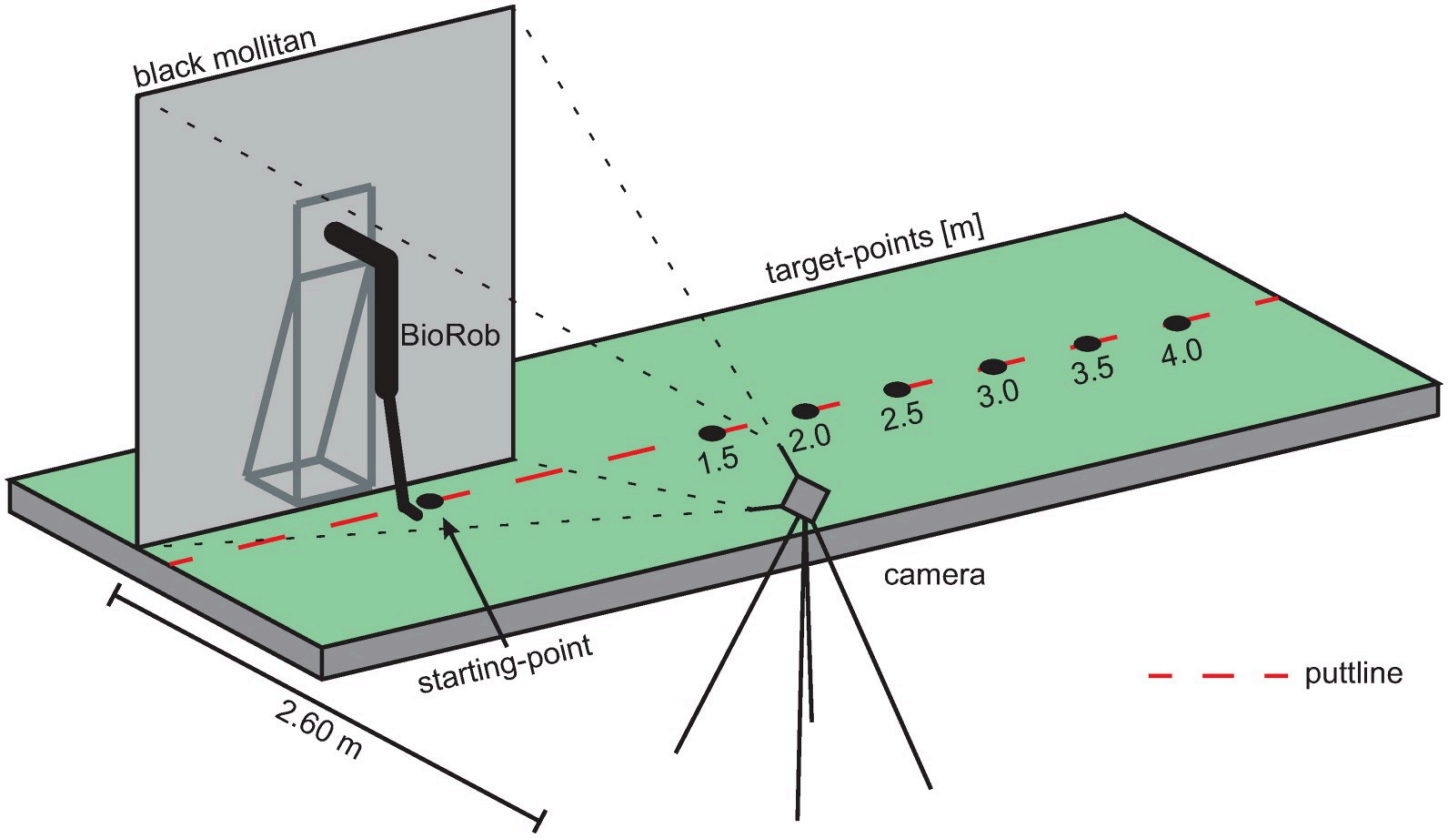
Video recording setup. Schematic representation of technical arrangement for the recording of the video material.

**Video material**. The robot performed putting movements over 6 different putting distances (1.5, 2.0, 2.5, 3.0, 3.5, and 4.0 m) on an artificial putting green. The robot motions were recorded using a Camcorder (Sony FDRAX33) with 50 frames per second. The camera was positioned at a distance of 2.6 m from the ball, perpendicular to the putting direction. A black mollitan was used as a background, which also covered the mounting frame of the BioRob system (see Fig 3).

**Fig 4:**
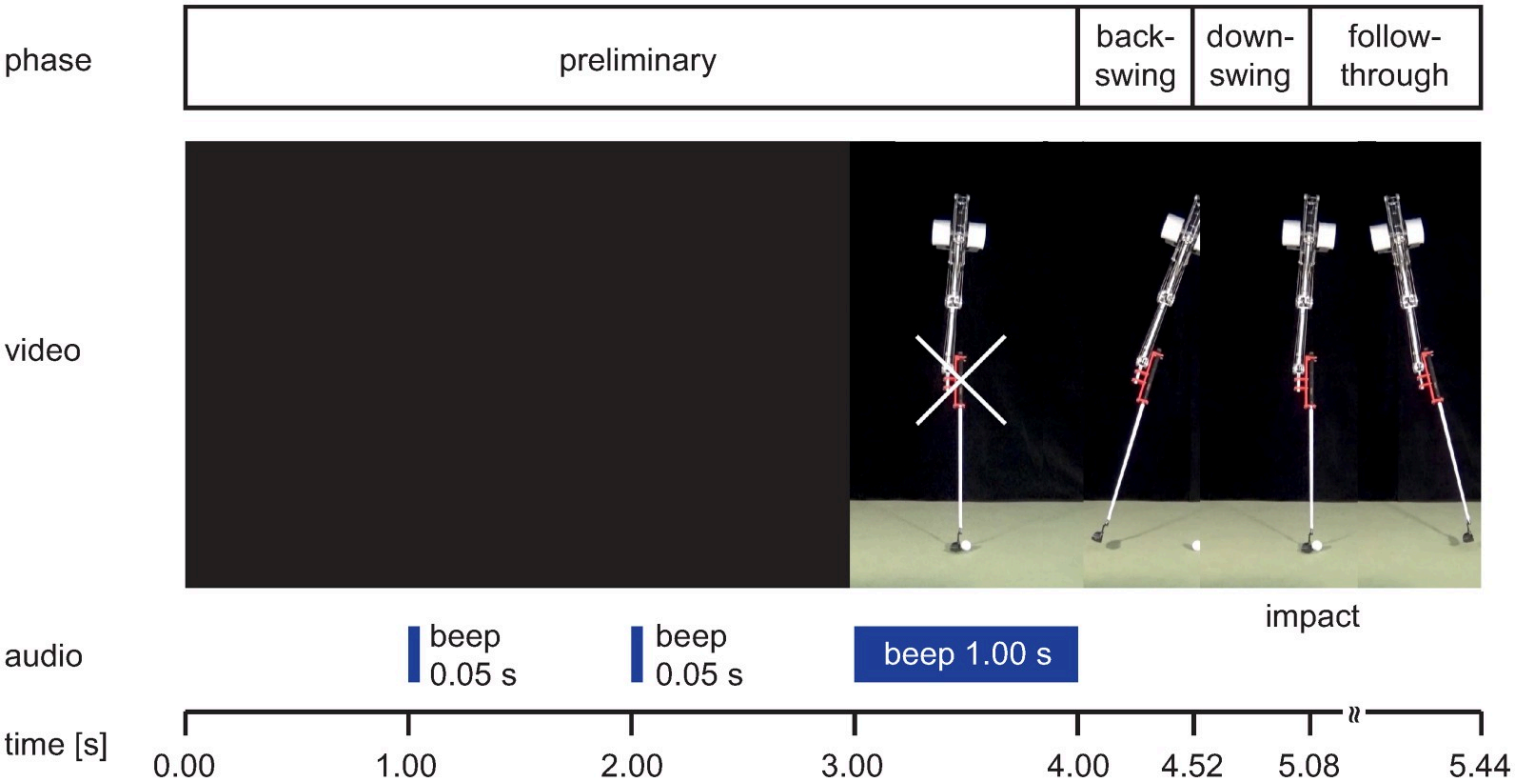
Video sequence in the full vision condition. Sequential presentation of a 2.0 m putt video in full vision condition with information about swing phase, audio signals and timing (video sequence see S1 Video).

**Fig 5:**
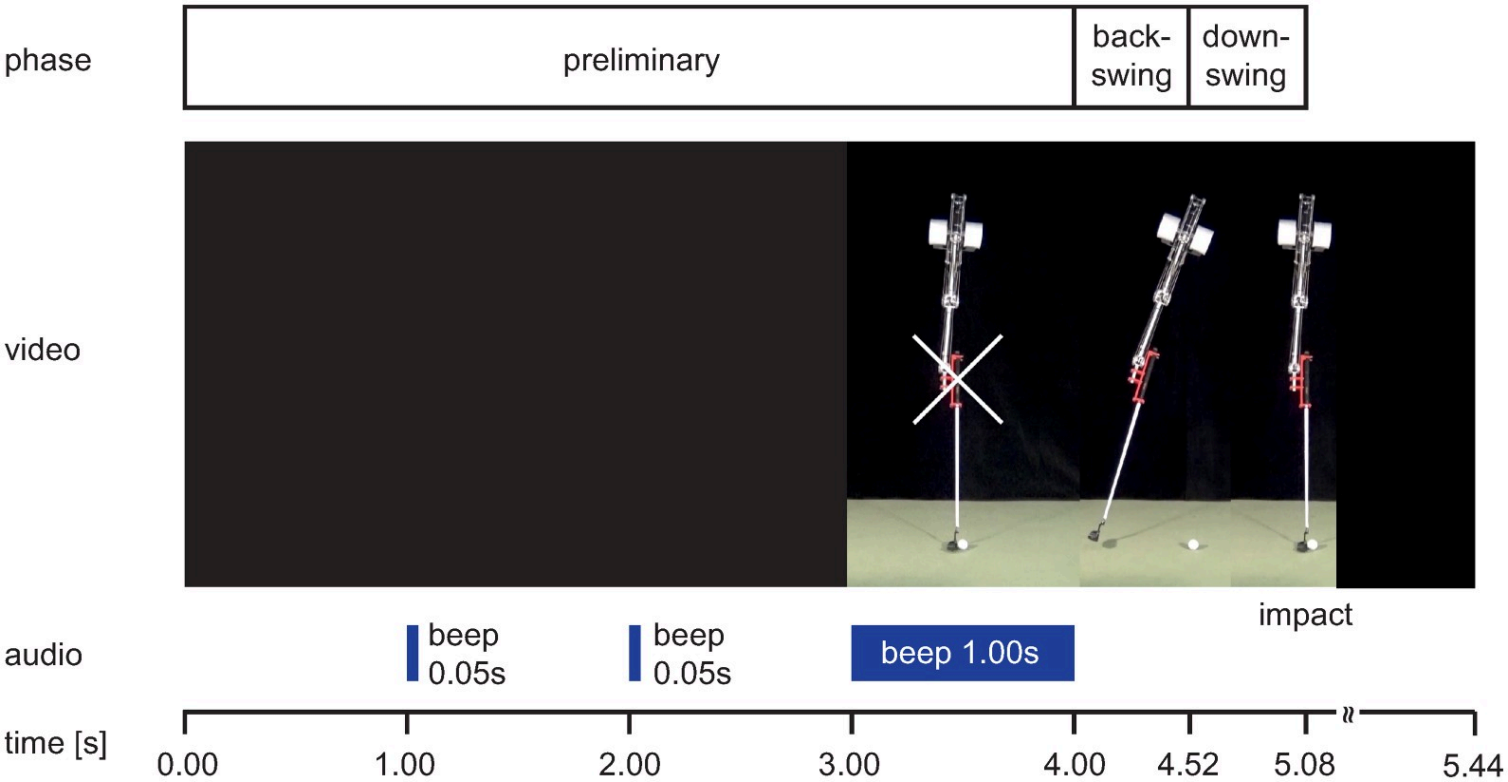
Video sequence in the incomplete vision condition. Sequential presentation of a 2.0 m putt video in incomplete vision condition with information about swing phase, audio signals and timing (video sequence see S2 Video).

The presented video material was produced with Adobe Creative Cloud Premiere Pro CC 2018 (Version 12.0.0). All 12 video scenes had the same basic structure (see Figs 4 and 5):

1. Preliminary phase: black screen (duration 3 s) with short beeps (duration 0.05 s) after 1 and 2 s, followed by a 1 s freeze frame of the robot in the starting position with a fixation cross centered on the handle and a 1 s beep;
2. Backswing phase: identical motion sequence from starting position to reversal point (duration 0.52 s). Regardless of the putting distance, velocity, joint angle, and reversal point were kept constant to avoid spatial stimuli in this phase;
3. Downswing phase: acceleration profiles from reversal to impact depending on putting distance;
4. Follow-through phase: rolling ball and club motion from impact until the ball passes the right boundary of the image.
  Note: The follow-through phase was presented only in the full vision condition.

**Stimuli description**. The duration of the different phases and the total duration of the video for the different putting distances are illustrated in Tables 3 and 4. In addition, the velocities of the club and the ball at the time of the impact are specified. The total duration of the preliminary (t_preliminary phase_) and backswing (t_backswing_) phases is the same (4 s) for all video sequences. The duration of the complete video sequences is reduced by 0.34 s from 5.54 s for a putting distance of 1.5 m to 5.20 s for a putting distance of 4.0 m. As the putting distance increases, the total duration of the downswing and follow-through phase decreases by 0.34 s from 1.02 s (downswing: 0.58 s; follow-through: 0.44 s) to 0.68 s (downswing: 0.46 s; follow-through: 0.22 s).

**Table 3:** Temporal and kinematic differences between the 6 putting distances under full and incomplete vision conditions.

Distance [m]	Full and incomplete vision condition				
	t_Total-I-RCHB_ [s]	t_preliminary phase_ [s]	t_backswing_ [s]	t_downswing_ [s]	v_C-impact_ [m/s]
1.5	5.10	4.00	0.52	0.58	0.8
2.0	5.08	4.00	0.52	0.56	0.9
2.5	5.06	4.00	0.52	0.54	1.0
3.0	5.04	4.00	0.52	0.52	1.2
3.5	5.02	4.00	0.52	0.50	1.5
4.0	4.98	4.00	0.52	0.46	1.7

t_Total-I-RCHB_ = total duration of the video in the incomplete vision condition; t_preliminary phase_ = duration of the preliminary phase; t_backswing_ = duration of the backswing phase; t_downswing_ = duration of the downswing phase; v_C-impact_ = resulting velocity of the club head at the impact.

**Table 4:** Temporal and kinematic differences between the 6 putting distances under full vision condition only.

Distance [m]	Full vision condition		
	t_Total-F-RCHB_ [s]	t_follow-through_ [s]	v_B-Impact_ [m/s]
1.5	5.54	0.44	1.4
2.0	5.44	0.36	1.6
2.5	5.38	0.32	1.9
3.0	5.32	0.28	2.0
3.5	5.26	0.24	2.3
4.0	5.20	0.22	2.5

t_Total-F-RCHB_ = total duration of the video in the full vision condition; t_follow-through_ = duration of follow-through phase; v_B-Impact_ = resulting velocity of the ball after the impact.

**Fig 6:**
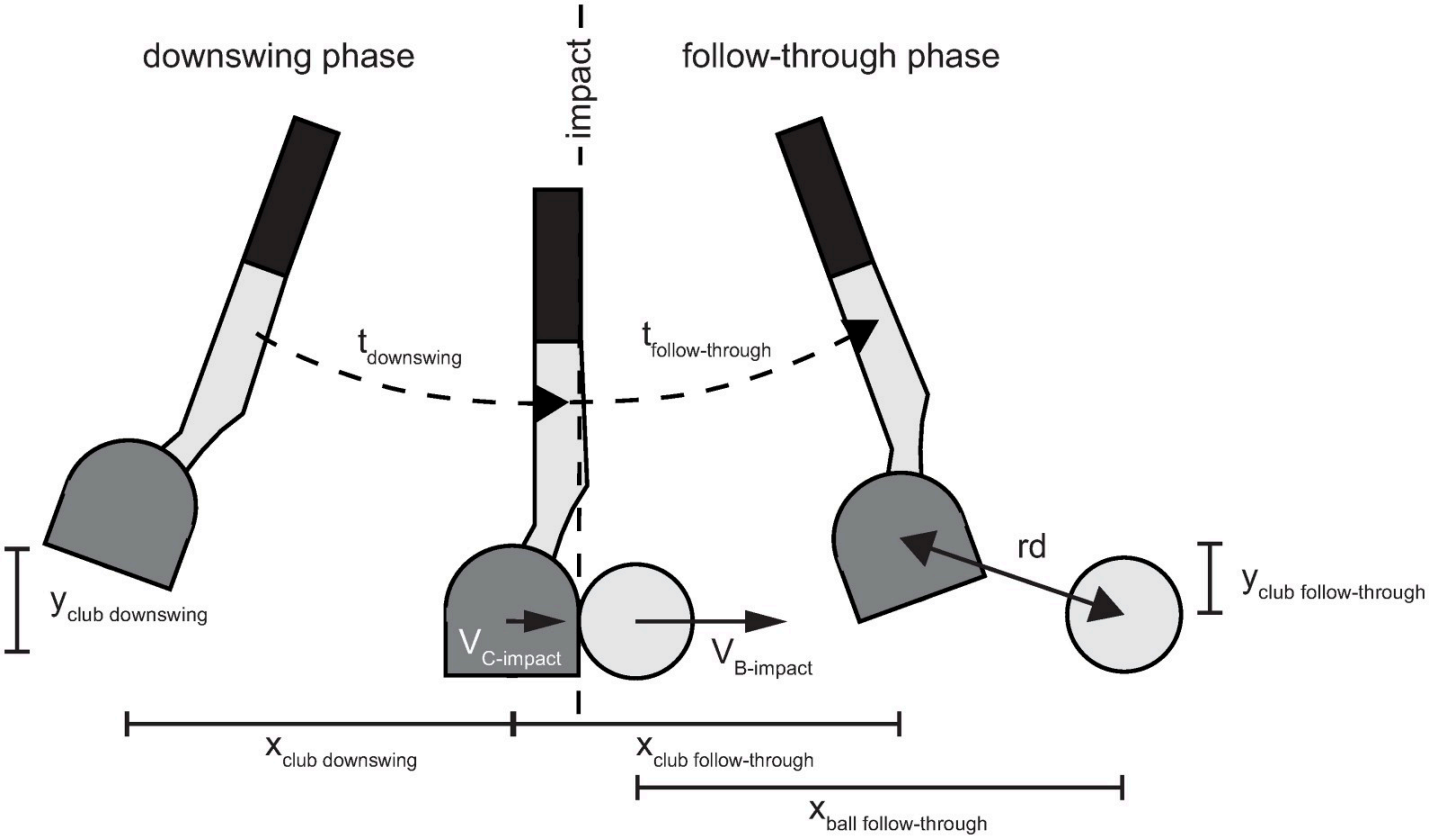
Stimuli description. Schematic representation of t_downswing_ = duration of the downswing phase; v_C-impact_ = resulting velocity of the club head before the impact; x_club downswing_ = distance covered by the club head in x-direction in the downswing phase; y_club downswing_ = distance covered by the club head in y-direction in the downswing phase; v_B-Impact_ = resulting velocity of the ball after the impact; t_follow-through_ = duration of follow-through phase; x_club follow-through_ = distance covered by the club head in x-direction in the follow-through phase; y_club follow-through_ = distance covered by the club head in y-direction in the follow-through phase; x_ball follow-through_ = distance covered by the ball in x-direction in the follow-through phase; rd = radial distance between club head and ball.

**Fig 7:**
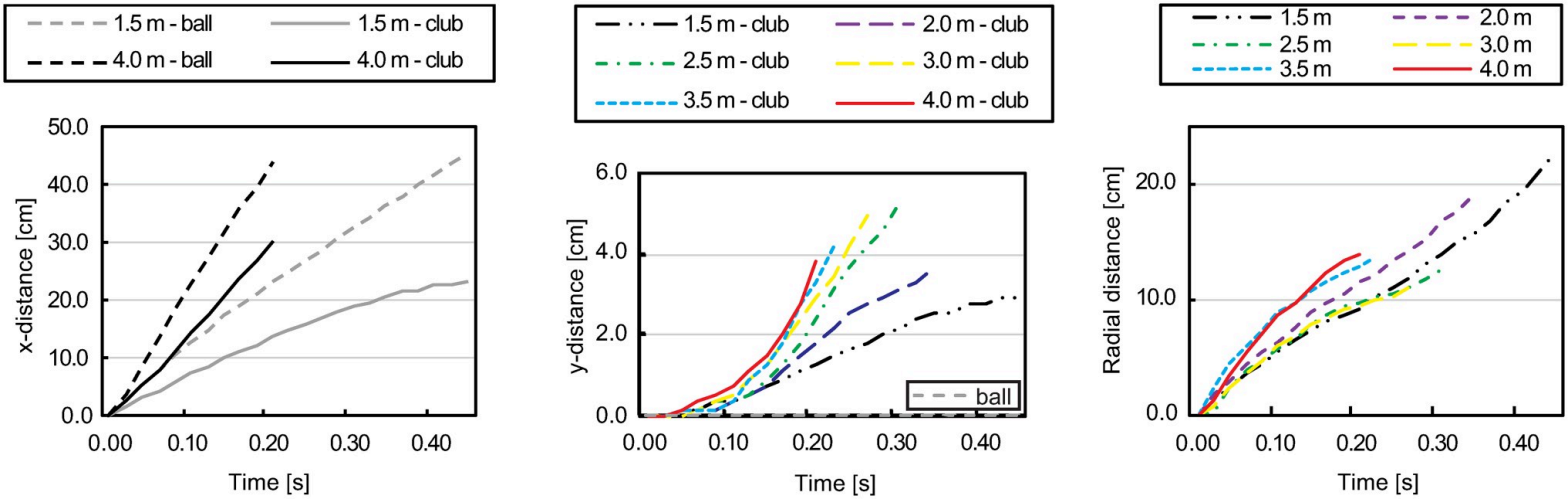
Schematic representation of stimuli in the follow-through phase. **Left**: Covered distance of ball and racket in x-direction after impact for putting distances 1.5 m and 4.0 m. **Center**: Covered distance of ball and racket in y-direction after impact for all putting distances. **Right**: Schematic representation of radial distance (rd) between club and ball. Note: Plots show real values measured with actual robot movements which were not perfectly smooth.

The velocity of the club head at impact and the ball velocity immediately after impact increase with increasing putting distances. In the incomplete vision condition, there is restricted information depending on the different putting distances, these are the duration of the downswing phase (t_downswing_), the total duration of the video (t_Total-I-RCHB_) and the resulting velocity of the club head before the impact (v_C-impact_). Additional spatio-temporal information is available in the full vision condition, i.e., the velocity of the ball after impact (v_B-impact_), duration of follow-through phase (t_follow-through_), and total duration (t_Total-F-RCHB_ and Fig 6). Furthermore, three spatial stimuli are delivered (see Table 5):

1. The distance covered by the ball and the club head in the x-direction after impact, as illustrated in Fig 7 (left) for the putting distances of 1.5 m and 4.0 m. In both cases, similar ball-to-club head relationships exist.
2. The distance of the club in the y-direction after impact. The covered distance of the club head varies depending on the putting distance between 3.0 cm and 5.3 cm (see Fig 7 center).
3. The radial distance between ball and club head after impact (see Fig 7 right).

**Table 5:** Comparison of available spatio-temporal information under full (F-RCHB) and incomplete (I-RCHB) vision condition.

Phase	Information	F-RCHB	I-RCHB
Downswing	t_downswing_	X	X
	t_Total_	X	X
	x_club downswing_	X	X
	y_club downswing_	X	X
	v_C-impact_	X	X
Follow-through	v_B-impact_	X	
	t_follow-through_	X	
	x_club follow-through_	X	
	y_club follow-through_	X	
	x_ball follow-through_	X	
	rd	X	

t_downswing_ = duration of the downswing phase; t_Total_ = total duration of the video; x_club downswing_ = distance covered by the club head in x-direction in the downswing phase; y_club downswing_ = distance covered by the club head in y-direction in the downswing phase; v_C-impact_ = resulting velocity of the club head before the impact; v_B-Impact_ = resulting velocity of the ball after the impact; t_follow-through_ = duration of follow-through phase; x_club follow-through_ = distance covered by the club head in x-direction in the follow-through phase; y_club follow-through_ = distance covered by the club head in y-direction in the follow-through phase; x_ball follow-through_ = distance covered by the ball in x-direction in the follow-through phase; rd = radial distance between club head and ball in the follow-through phase.

Fig 7 shows a schematic representation of these additional stimuli.

**Fig 8:**
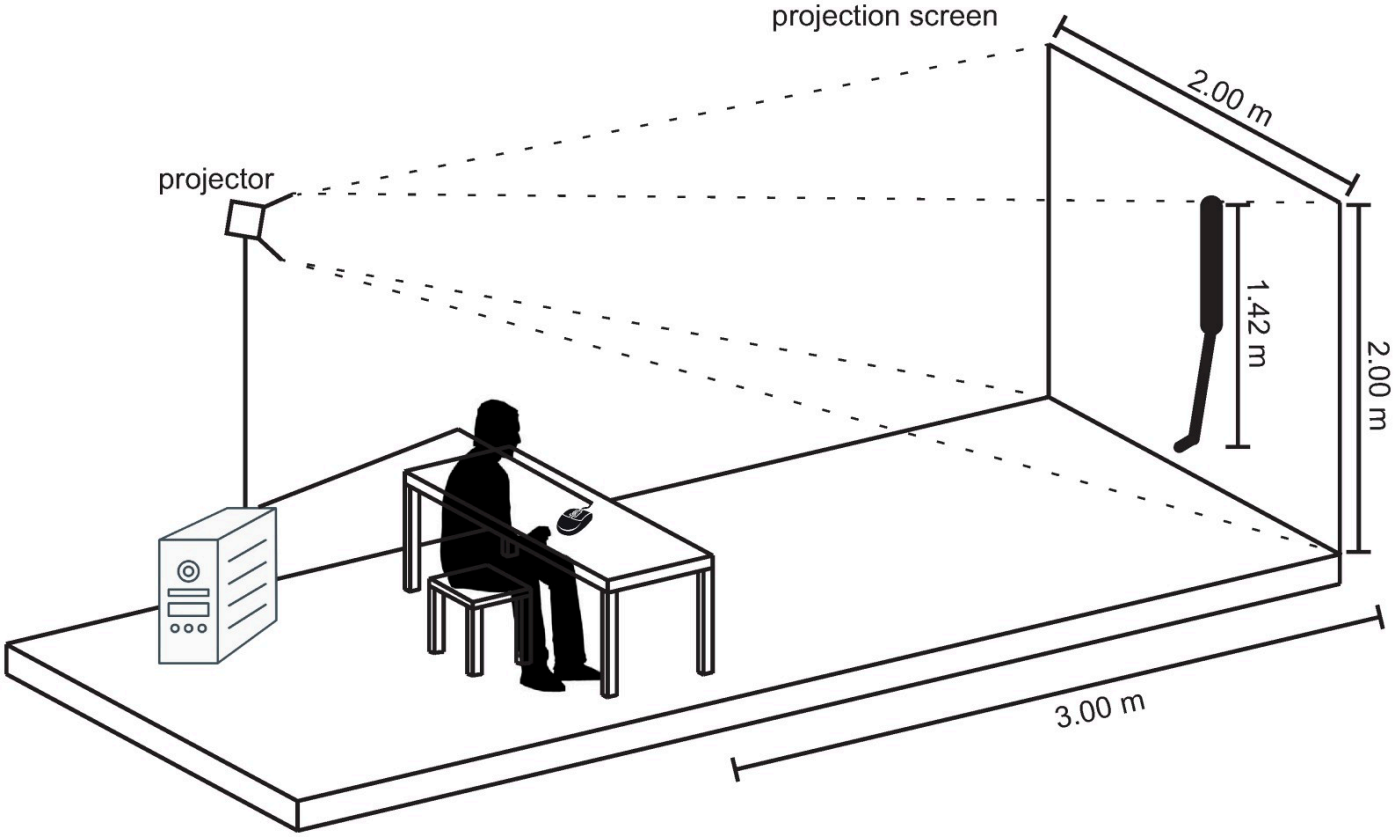
Experimental setup. Schematic representation of the experimental setup with the projection screen and the position of the participant.

**Experimental setup**. The video sequences were presented by a self-developed computer program. Video clips were displayed by a projector (EPSON EB-1860, resolution: 1024 x 768 px) in original size at the end of the artificial putting green with no sound. The BioRob system was projected in its real size, i.e., 1.42 m. Participants watched the video sequences from a distance of 3.0 m while sitting at a table (see Fig 8).

Each of the 12 video sequences was shown four times to the participants in randomized order (total of 48 clips). Upon completion of each sequence, a visual analog scale (0.0 to 6.0 m) was presented to the participants by the computer program. The scale was displayed with a width of 1.050 px (spatial resolution: 0.57 cm/px). Participants provided their length prediction by clicking at the respective point on the scale with the mouse cursor.

Following the estimation of the putting distance, participants provided the confidence of their decision on a five-point scale (very unsure, unsure, undecided, sure, and very sure). In addition to the prediction of the putting distance and the confidence, the response time, i.e. time elapsed between the end of the video presentation and the final click on the distance scale, was also recorded. After assessment, the next video was started by clicking a button. All data was stored by the computer program in one file for each participant.

**Procedure**. First, the participants were introduced to the laboratory and the experimental setup by the experimenter. After this introduction, all participants received an informed consent document and a participant questionnaire. After signing the consent and completing the questionnaire, the test software was presented to the participants and the experimental procedure including the collected data (e.g. prediction, reaction time) was described (Fig 8). The participants read the instructions and any questions were answered by the experimenter. After the introductory phase, the participants started the experiment autonomously according to the procedure explained in the previous section. After completion of the test program, the participants were debriefed.

**Data processing and analysis**. Based on the predicted putting distance, constant error (CE), absolute error (AE) and the variable error (VE) were calculated [38, p.55-64]. The CE measures the average error of responding and thus the accuracy [38, p.57]:

$$\begin{array}{c} \text{CE}=\frac{\sum_{i=1}^{n}\left(x_{i}-T\right)}{n} \end{array}$$ (1)

The AE measures the overall accuracy and is the average absolute error between the predicted distance and the real put distance without regard to the direction [38, p.59]:

$$\begin{array}{c} \text{AE}=\frac{\sum_{i=1}^{n}\left|x_{i}-T\right|}{n} \end{array}$$ (2)

The VE is the variability about the mean response and measures the inconsistency in predicting [38, p.58]:

$$\begin{array}{c} \text{VE}=\sqrt{\frac{\sum_{i=1}^{n}{\left(x_{i}-\bar{x}\right)}^{2}}{n}} \end{array}$$ (3)

A two-way ANOVA with repeated measures was calculated with the two factors of putting distance (6 distances) and vision condition (full versus incomplete). Wilcoxon tests were applied for follow-up analysis. Bonferroni corrections were applied to multiple comparisons. All statistical analyses were calculated using SPSS 24 (SPSS Inc., Chicago, USA). Level of significance was set a priori to 0.05.

### Results

The following section describes the results of the experiment 1. A dataset with the raw data and the calculated values can be found in the supporting information (see S1 Dataset).

#### Prediction of the putting distance

**Fig 9:**
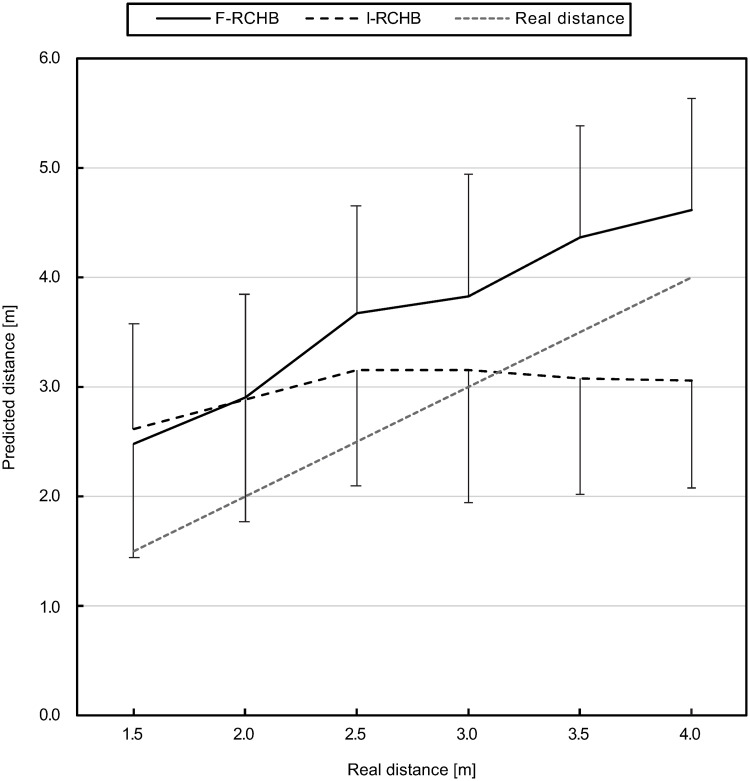
Predicted putting distance. Mean and standard deviation of the predicted putting distance under full (F-RCHB) and incomplete (I-RCHB) vision condition.

Fig 9 (descriptive statistics see S1 Table) show the means and standard deviations of the predicted putting distance for the six real putting distances under the two experimental conditions. The prediction of the putting distance differs under the two conditions with the exception of the putting distance of 2.0 m. Under the full vision condition, all distances are overestimated and the estimated putting distance increases with increasing distance of the putts presented to the participants. The distance prediction under the incomplete vision condition does not correspond to the real distance; estimations show a slight increase (by 0.54 m) in the range of the real putting distance from 1.5 to 2.5 m. In the distance range from 2.5 to 4 m, the prediction remains constant (3.15 to 3.05 m). Shorter distances (1.5—3.0 m) are overestimated, while longer distances (3.5—4.0 m) are underestimated.

The two-factor ANOVA with repeated measures (6 distances: 1.5, 2.0, 2.5, 3.0, 3.5, and 4.0 m; 2 vision conditions: full and incomplete vision) revealed significant main effects of vision condition and distance as well as a significant interaction effect (see Table 6).

**Table 6:** Results of the two-factor ANOVA with repeated measures (6 distances; 2 vision conditions) for the predicted putting distance. Corrected by Greenhouse-Geisser *ϵ*.

Factor	df1	df2	F	p	*η*^2^_*p*_
Vision condition	1.00	19.00	35.86	<.001	.654
Distance	2.35	44.64	50.47	<.001	.726
Vision condition *x* distance	3.78	71.85	42.90	<.001	.693

A follow-up analysis using a Wilcoxon test with Bonferroni correction (descriptive statistics see S1 Table) revealed significant differences between the vision conditions at all distances, except for the short putting distances of 1.5 and 2.0 m.

#### Constant error (CE)

**Fig 10:**
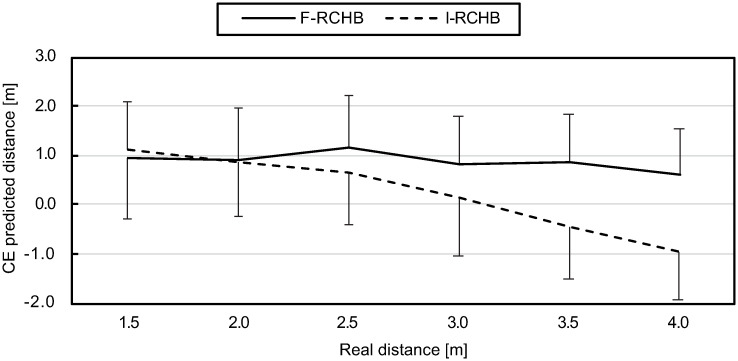
Constant error. Mean and standard deviation of the constant error of predicted putting distance under full (F-RCHB) and incomplete (I-RCHB) vision condition.

The constant error indicates the accuracy of the prediction of the putting distance with respect to the actual length of the putt, i.e., the average error. Under the incomplete vision condition the constant error decreases from 1.11 m (1.5 m) to 0.15 m (3.0 m) with increasing distances for small and medium distances. For longer distances, the sign changes and the constant error increases to -0.95 m (4.0 m). In contrast, the constant error under the constant vision condition is fairly constant (within a range of 0.62 to 1.16 m), showing a slightly decreasing trend with increasing putting distance (see Fig 10, detail table see S1 Table).

The two-factor ANOVA with repeated measures (6 distances: 1.5, 2.0, 2.5, 3.0, 3.5, and 4.0 m; 2 vision conditions: full and incomplete vision) revealed significant main effects of vision condition and distance as well as a significant interaction effect (see Table 7).

**Table 7:** Results of the two-factor ANOVA with repeated measures (6 distances; 2 vision conditions) for the constant error of the predicted putting distance. Corrected by Greenhouse-Geisser *ϵ*.

Factor	df1	df2	F	p	*η*^2^_*p*_
Vision condition	1.00	19.00	35.86	<.001	.654
Distance	2.35	44.64	45.49	<.001	.705
Vision condition *x* distance	3.78	71.85	42.90	<.001	.693

A follow-up analysis using a Wilcoxon test with Bonferroni correction (descriptive statistics see S1 Table) revealed significant differences between the vision conditions at all distances, except for short putting distance of 1.5 and 2.0 m.

#### Variable error (VE)

**Fig 11:**
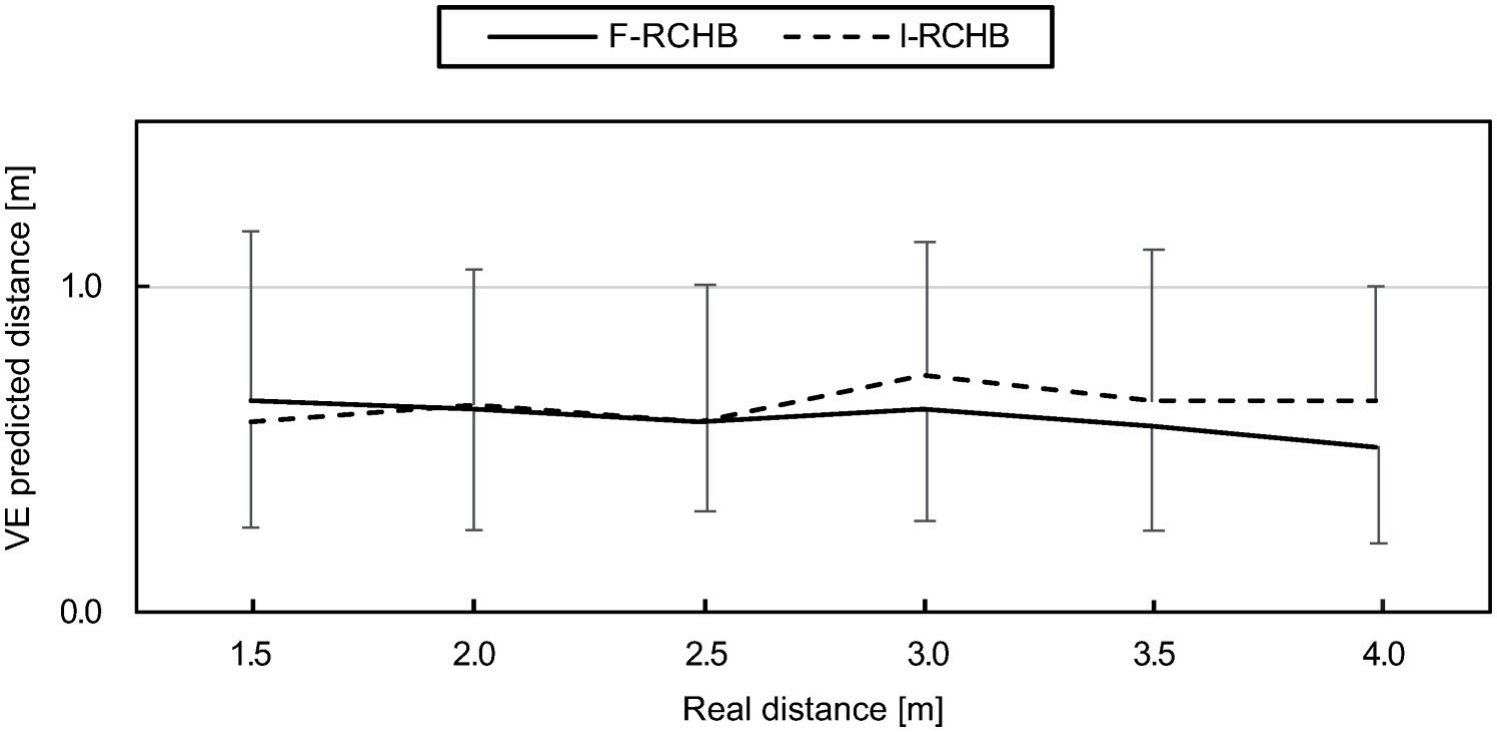
Variable error. Mean and standard deviation of the variable error of predicted putting distance under full (F-RCHB) and incomplete (I-RCHB) vision condition.

Variable error indicates the consistency of the estimate of the putting distance, i.e., the variability of the participants estimates around the mean of prediction of the putting distance. As can be seen in Fig 11 (descriptive statistics see S1 Table), variable error is rather constant under the full vision condition, whereas it slightly increases with increasing putting distance under the incomplete vision condition.

The two-factor ANOVA with repeated measures (6 distances: 1.5, 2.0, 2.5, 3.0, 3.5, and 4.0 m; 2 vision conditions: full and incomplete vision) revealed no significant main effects of vision condition and distance or interaction effect (see Table 8).

**Table 8:** Results of the two-factor ANOVA with repeated measures (6 distances; 2 vision conditions) for the variable error of the predicted putt length. Corrected by Greenhouse-Geisser *ϵ*.

Factor	df1	df2	F	p
Vision condition	1.00	19.00	1.09	.310
Distance	3.56	67.70	.454	.748
Vision condition *x* distance	3.78	71.74	.808	.581

#### Absolute error (AE)

**Fig 12:**
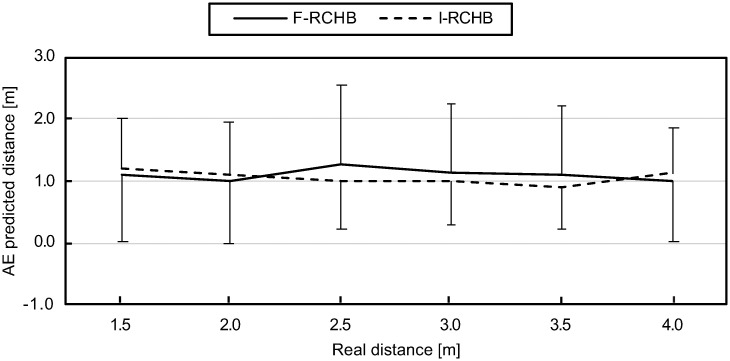
Absolute error. Mean and standard deviation of the absolute error of predicted putting distance under full (F-RCHB) and incomplete (I-RCHB) vision condition.

The absolute error measure is the absolute average between the prediction and the real distance. Under the incomplete vision condition, the absolute error initially decreases with increasing real putting distance and increases again at 4.0 m. Under the complete vision condition, the absolute error decreases from the real putting distance of 1.5 to 2.0 m, increases at a putting distance of 3.0 m and then decreases to its minimum at 4.0m (Fig 12, descriptive statistics see S1 Table).

The two-factor ANOVA with repeated measures (6 distances: 1.5, 2.0, 2.5, 3.0, 3.5, and 4.0 m; 2 vision conditions: full and incomplete vision) revealed no significant main or interaction effects (see Table 9).

**Table 9:** Results of the two-factor ANOVA with repeated measures (6 distances; 2 vision conditions) for the absolute error of the predicted putt length. Corrected by Greenhouse-Geisser *ϵ*.

Factor	df1	df2	F	p
Vision condition	1.00	19.00	.174	.681
Distance	2.30	43.78	.356	.732
Vision condition *x* distance	2.50	47.45	2.05	.129

#### Confidence of prediction

**Fig 13:**
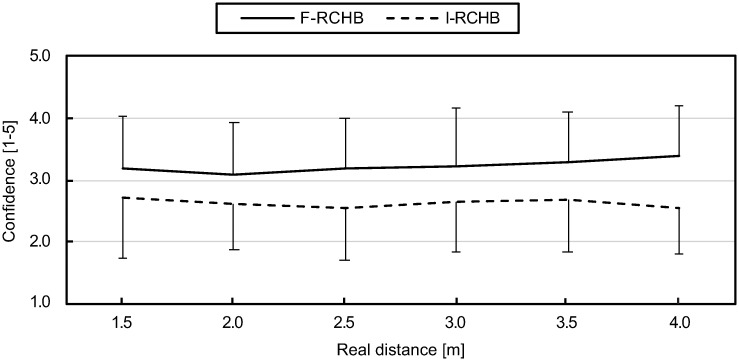
Confidence of prediction. Mean and standard deviation of the confidence of prediction depending on the real putting distance under full (F-RCHB) and incomplete (I-RCHB) vision condition.

Confidence was recorded on a five-point scale with the values: (1) very unsure, (2) unsure, (3) undecided, (4) sure, and (5) very sure. Fig 13 (descriptive statistics see S1 Table) show the means and standard deviations of the confidence of the predicted putting distance of all participants for the six real putting distances under the two experimental conditions. The confidence of the predicted putting distance differs under the two conditions over all distances. Under the complete vision condition, confidence of prediction was higher and shows a slight increase with increasing putting distance from 3.09 at 2.0 m to 3.40 at 4.0 m. Under the incomplete vision condition, the confidence reaches the highest value at the putting distance of 1.5 m (2.70), remains nearly constant and decreases at the putting distance of 4.0 m to the lowest value of 2.57.

The two-factor ANOVA with repeated measures (6 distances: 1.5, 2.0, 2.5, 3.0, 3.5, and 4.0 m; 2 vision conditions: full and incomplete vision) revealed a significant main effect of vision condition (see Table 10).

**Table 10:** Results of the two-factor ANOVA with repeated measures (6 distances; 2 vision conditions) for the confidence of the prediction. Corrected by Greenhouse-Geisser *ϵ*.

Factor	df1	df2	F	p	*η*^2^_*p*_
Vision condition	1.00	19.00	41.34	<.001	.685
Distance	3.34	63.41	1.46	.232	.071
Vision condition *x* distance	3.56	67.66	2.10	.098	.100

#### Response time

**Fig 14:**
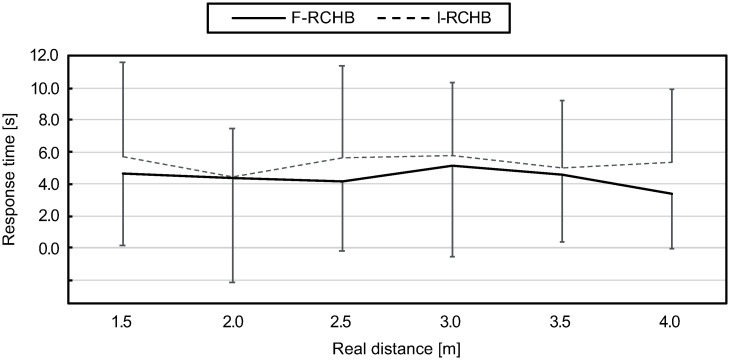
Response time. Mean and standard deviation of the response time depending on the real putting distance under full (F-RCHB) and incomplete (I-RCHB) vision condition.

The response time was measured as the time between the end of the video sequence and the final mouse click on the meter scale. Fig 14 (descriptive statistics see S1 Table) show the means and standard deviations of the response time of all participants for the six real putting distances under the two experimental conditions. The response times in the two conditions differ over all distances and are lower under the complete vision condition. With increasing distance, the response time under the complete vision condition at shorter distances decreases from 4.64 s (1.5 m) to 4.17 s (2.5 m) followed by an increase to 5.13 s (3.0 m). For longer distances, the response time decreases again as the distance increases, reaching its minimum at 3.38 s (4.0 m). Under the incomplete vision condition, the response time decreases from 5.70 s (1.5 m) to 4.47 s (2.0 m) and increases to 5.61 and 5.77 s for medium distances (2.5 and 3.0 m). For long distances, the response time decreases from the medium distances to 4.99 s (3.5 m) and 5.33 s (4.0 m).

The two-factor ANOVA with repeated measures (6 distances: 1.5, 2.0, 2.5, 3.0, 3.5, and 4.0 m; 2 vision conditions: full and incomplete vision) revealed a significant main effect of vision condition (see Table 11).

**Table 11:** Results of the two-factor ANOVA with repeated measures (6 distances; 2 vision conditions) for the response time. Corrected by Greenhouse-Geisser *ϵ*.

Factor	df1	df2	F	p	*η*^2^_*p*_
Vision condition	1.00	19	50.17	<.001	.725
Distance	3.60	68.39	2.54	.053	.118
Vision condition *x* distance	3.02	57.39	1.14	.342	.056

### Discussion of experiment 1

The results show that participants were able to predict the putting distance of a robot putt under the complete vision condition. However, all six putting distances were overestimated by the participants, a linear course of the predicted putting distances is shown with increasing real putting distance. This systematic overestimation supports the predictions derived from the FLE. However, contrary to hypothesis 1, prediction error (CE) does not increase, but rather decreases with increasing putting distance. In contrast to the full vision condition, the participants could not predict the putting distance under the incomplete vision condition. The predicted values seem to be randomly chosen values that show a tendency to the centre of the prediction scale (0 to 6m) at 3.00 m. The confidence data support the prediction results. The visibility of the follow-through-phase was found to have a decisive influence on the quality of the prediction of the putting distance. This leads to the conclusion that the additional stimuli available under the full vision condition have a high relevance for the prediction of putting movements. It remains open what influence individual elements (e.g. club and robot) have on the prediction of the putting distance. Whereas hypothesis 2 (different prediction depending on vision condition) was confirmed, there was no evidence for hypothesis 1 (increasing error with increasing putting distance). Ex post calculations of power using the software G*Power 3.1.9.4 [39] revealed that experiment 1 was over-powered (1.0) regarding hypothesis 2 (Protocol of power analysis see S1 File). Power analysis for hypothesis 1 does not make sense due to the decrease of error, which is contrary to hypothesis 1.

## Experiment 2—Prediction and visibility manipulation

This experiment is based on the results of experiment 1. In addition to a replication of the previous results (hypotheses 1 and 2), the influence of the visibility of the ball, club and robot after the impact on the prediction of the putting distance is investigated (spatial occlusion; hypothesis 3). In this experiment, video sequences of robot putts at three different distances are presented to the participants. For each distance, videos are shown under four different visual conditions, i.e. full vision condition (F-RCHB), incomplete vision condition (I-RCHB), full vision condition with visible robot, club and club head in the follow-through-phase (F-RCH), and full vision condition with visible ball in the follow-through-phase (F-B).

In the following, only differences in materials and methods compared to experiment 1 are presented.

### Materials and methods

#### Participants

Nineteen healthy students (11 males and 8 females), aged 19 to 36 years, volunteered to participate in the experiment. Inclusion criteria was no previous experience with perceptual studies. Demographic data are presented in Table 12. Whereas the results from Experiment 1 resulted in an optimal sample size of 4 participants (hypothesis 2), estimated sample size for testing hypothesis 3 was 12 participants [39](Protocol of power analysis see S2 File). Taking into account the “winner’s curse phenomenon”, it is expected that the true effect size of experiment 1 regarding hypothesis 2 will be smaller [40]. Therefore, the replication experiment will test a similar number of participants (N = 19) as in Experiment 1 [40].

**Table 12:** Demographic data of the participants (mean±SD).

	n	Age [yr]	Height [cm]	Body mass [kg]	Handedness [left | right]
Female	8	24.0±3.2	166.0±6.6	59.0±5.3	3 | 5
Male	11	26.0±4.8	182.0±5.5	83.0±8.7	0 | 12
Total	19	25.0±4.3	176.0±9.8	73.0±14.0	3 | 17

Table 13 shows the information provided by participants regarding their previous experience in four different groups of activities (see Experiment 1—Participants). This experiment also conducted in accordance with the declaration of Helsinki in its latest version. All participants provided written informed consent before participation. The Ethical Committee of Technische Universität Darmstadt (TU Darmstadt) specifically approved this experiment.

**Table 13:** Experience in selective sports and computer games (mean±SD).

	Golf, field hockey and similar			Returning games			Ball games			Computer games		
	n	years	h/wk	n	years	h/wk	n	years	h/wk	n	years	h/wk
Female	3	3.8±3.8	3.5±3.2	4	8.3±8.8	1.5±0.5	5	5.2±4.2	4.6±3.0	5	11.0±6.5	3.0±1.4
Male	5	4.9±3.4	8.4±7.8	7	9.0±5.8	3.9±3.0	11	16.2±8.4	7.8±9.2	11	12.1±6.3	10.7±10.1
Total	8	4.5±3.3	6.6±6.6	11	8.7±6.6	3.0±2.6	16	12.8±8.9	6.8±7.9	16	11.8±6.2	7.7±9.3

Means and SD were only calculated for participants reporting experience.

#### Apparatus and task

Based on the video recordings produced for Experiment 1 (see Experiment 1—Materials and methods), additional scenes for the conditions F-RCH (full vision—robot, club, and club head visible) and F-B (full vision—robot and ball) were created for the three distances (2.0, 3.0, and 4.0 m). All 12 video scenes have the same basic structure (see Figs 4 and 5):

1. Preliminary phase
2. Backswing phase
3. Downswing phase
4. Follow-through phase: rolling ball and club motion from impact until the ball passes the right boundary of the image (see Fig 4). Note: In the F-RCH/F-B only the club and robot/ball were presented. The follow-through-phase was not presented in the I-RCHB.

In addition to the video sequences of the distance of 2.0 m in conditions F-RCHB (S1 Video) and I-RCHB (S2 Video), the video sequences of conditions F-RCH (S3 Video) and F-B (S4 Video) are available as supporting information.

**Stimuli description**. Since the same video sequences were used as in experiment 1, the possible stimuli are analogous to this experiment (see Table 14).

**Table 14:** Comparison of available spatio-temporal information in F-RCHB, I-RCHB, F-RCH, and F-B. Explanations: see text.

Phase	Information	F-RCHB	I-RCHB	F-RCH	F-B
Downswing	*t*_downswing_	X	X	X	X
	*t*_Total_	X	X	X	X
	x_club downswing_	X	X	X	X
	y_club downswing_	X	X	X	X
	*v*_C-impact_	X	X	X	X
Follow-through	*v*_Bimpact_	X			X
	*t*_follow-through_	X		X	X
	*x*_club follow-through_	X		X	
	*y*_club follow-through_	X		X	
	*x*_ball follow-through_	X			X
	*rd*	X			

t_downswing_ = duration of the downswing phase; t_Total_ = total duration of the video; x_club downswing_ = distance covered by the club head in x-direction in the downswing phase; y_club downswing_ = distance covered by the club head in y-direction in the downswing phase; v_C-impact_ = resulting velocity of the club head before the impact; v_B-Impact_ = resulting velocity of the ball after the impact; t_follow-through_ = duration of follow-through phase; x_club follow-through_ = distance covered by the club head in x-direction in the follow-through phase; y_club follow-through_ = distance covered by the club head in y-direction in the follow-through phase; x_ball follow-through_ = distance covered by the ball in x-direction in the follow-through phase; rd = radial distance between club head and ball.

**Experimental setup**. The video sequences were presented in the same way as in experiment 1 (see Experiment 1—Apparatus and task) also without sound.

**Procedure**. The same procedure as in experiment 1 was used (see Experiment 1—Apparatus and task).

**Data processing and analysis**. Based on the predicted putting distance, absolute error (AE), constant error (CE) and the variable error (VE) were calculated [38]. A two-way ANOVA with repeated measures was calculated with the two factors of putting distance (3 distances) and vision condition (F-RCHB, I-RCHB, F-RCH, and F-B). Wilcoxon tests were applied for follow-up analysis. Bonferroni corrections were applied to multiple comparisons. All statistical analyses were calculated using SPSS 24 (SPSS Inc., Chicago, USA). Level of significance was set a priori to 0.05.

### Results

The following section describes the results of experiment 2. A dataset with the raw data and the calculated values can be found in the supporting information (see S2 Dataset).

#### Prediction of the putt length

**Fig 15:**
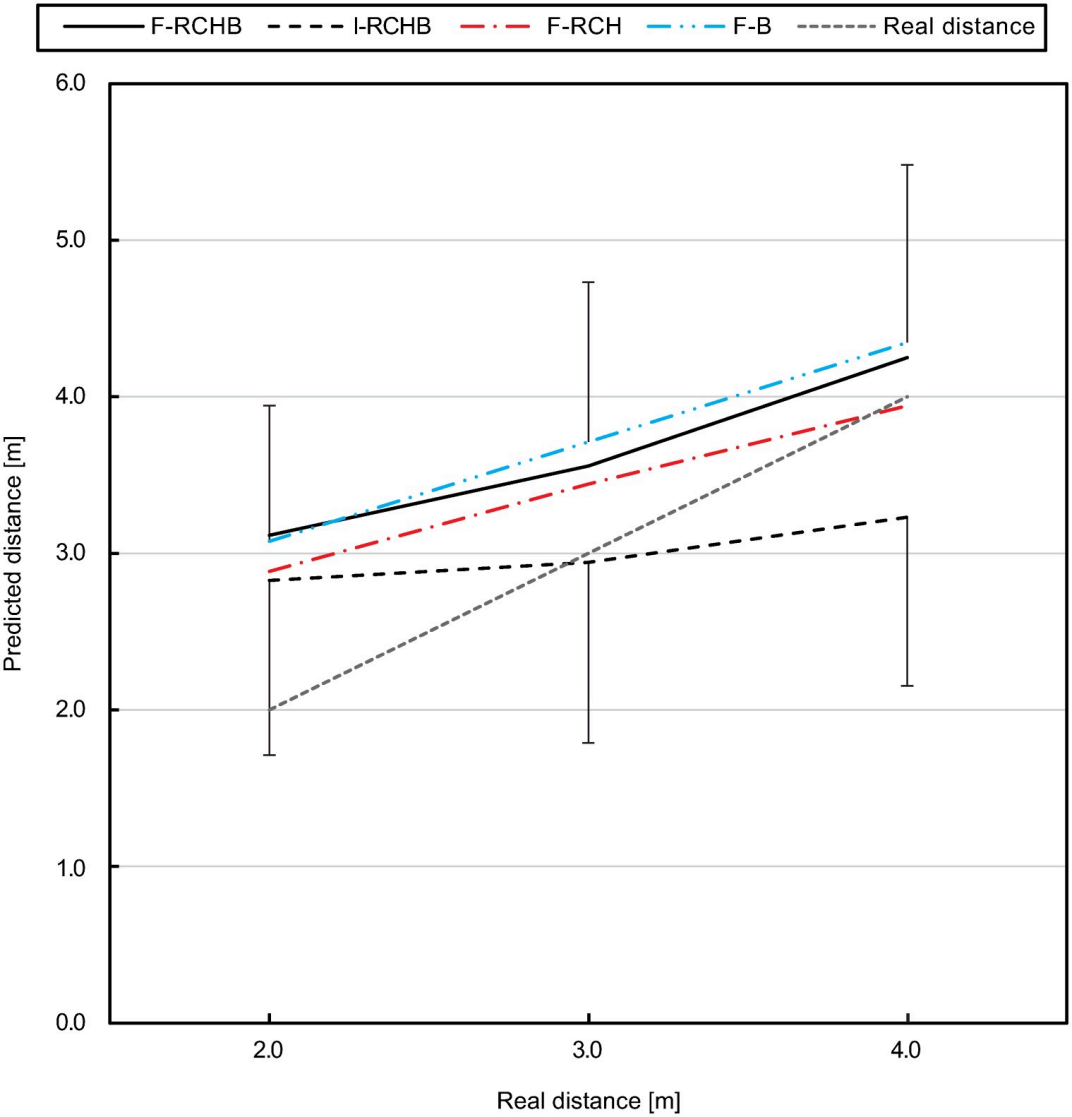
Predicted putting distance. Mean and standard deviation of the predicted putting distance under I-RCHB, F-RCHB, F-RCH, and F-B condition.

Fig 15 (descriptive statistics see S1 Table) show the means and standard deviations of the predicted putting distance of all participants for the three real putting distances under the four experimental conditions. Under the F-RCHB, F-RCH, and F-B conditions, the predicted putting distance increases with increasing real putting distance. All distances are overestimated under the F-RCHB and F-RCH conditions. Under the F-B condition, the distances of 2.0 and 3.0 m are overestimated, whereas the distance of 4m is slightly underestimated. The predicted distances under the I-RCHB condition show a small increase from 2.81 m (distance 2.0 m) to 3.22 m (distance 4.0 m). The distance of 2.0 m is overestimated and the distances of 3.0 and 4.0 m are underestimated.

The two-factor ANOVA with repeated measures (3 distances: 2.0, 3.0, and 4.0 m; 4 vision conditions: F-RCHB, I-RCHB, F-RCH, and F-B) revealed significant main effects of vision condition and distance as well as a significant interaction effect for predicted putt length (see Table 15).

**Table 15:** Results of the two-factor ANOVA with repeated measures (3 distances; 4 vision conditions) for the predicted putt length. Corrected by Greenhouse-Geisser *ϵ*.

Factor	df1	df2	F	p	*η*^2^_*p*_
Vision condition	1.92	34.50	12.04	<.001	.401
Distance	1.59	28.58	62.15	<.001	.775
Vision condition *x* distance	4.56	82.08	4.25	.002	.191

A follow-up analysis using a Wilcoxon test with Bonferroni correction (descriptive statistics see S1 Table) revealed no significant differences between the vision conditions at the real distance of 2.0 m. For the real distance of 3.0 m significant differences between the I-RCHB and the two manipulated vision conditions (F-RCH and F-B) are revealed. At a distance of 4.0 m there are significant differences between the incomplete (I-RCHB) and the three full (F-RCHB, F-RCH, and F-B) vision conditions.

#### Constant error (CE)

**Fig 16:**
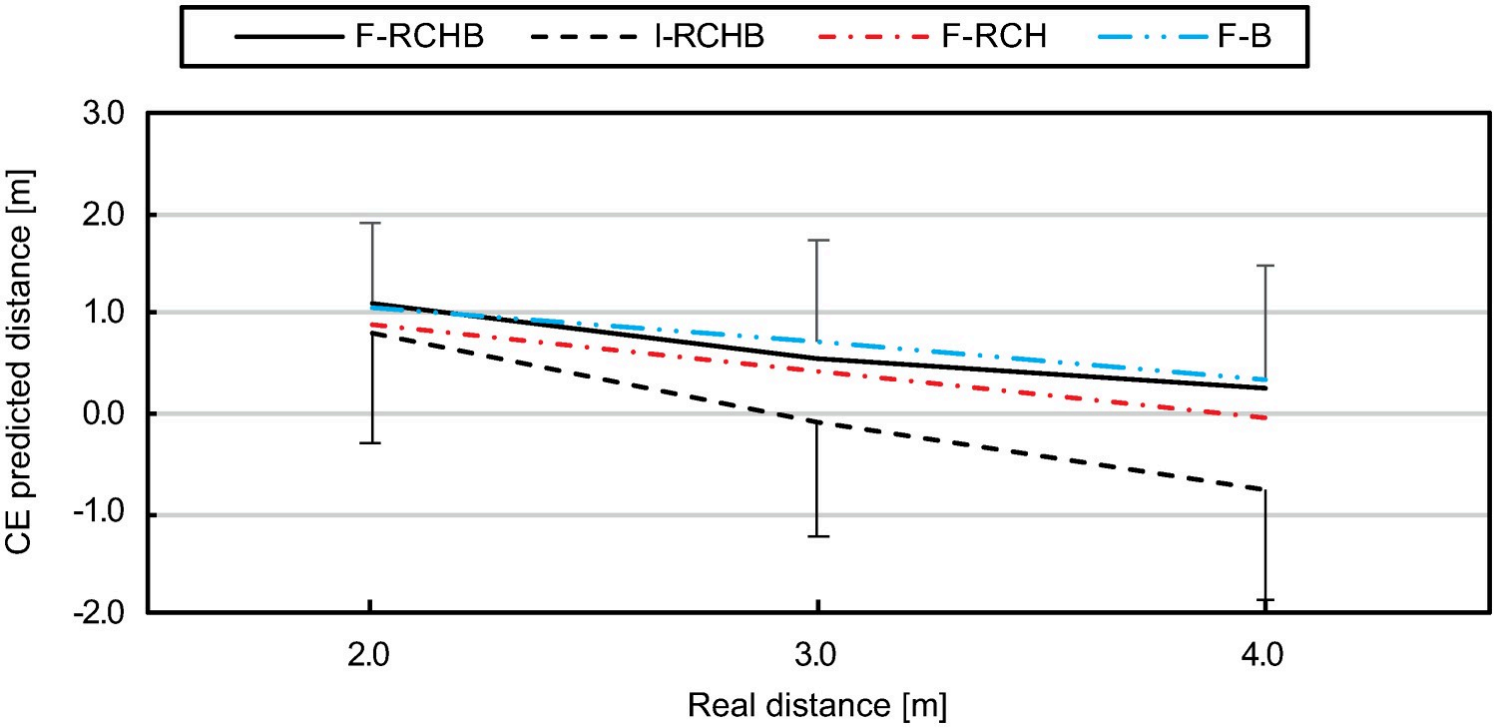
Constant error. Mean and standard deviation of the constant error of predicted putting distance under the I-RCHB, F-RCHB, F-RCH, and F-B conditions.

The constant error decreases with increasing real putting distance under all full vision conditions (see Fig 16, descriptive statistics see S1 Table). While the constant error under the F-RCHB, F-HCB, and F-B conditions decreases comparably, i.e., by 0.88m (F-RCHB), 0.94 (F-RCH) and 0.73 (F-B), the constant error under the I-RCHB condition changes sign from +0.81 to -0.77.

The two-factor ANOVA with repeated measures (3 distances: 2.0, 3.0, and 4.0 m; 4 vision conditions: F-RCHB, I-RCHB, F-RCH, and F-B) revealed significant main effects of vision condition and distance as well as a significant interaction effect (see Table 16).

**Table 16:** Results of the two-factor ANOVA with repeated measures (3 distances; 4 vision conditions) for the constant error of predicted putt length. Corrected by Greenhouse-Geisser *ϵ*.

Factor	df1	df2	F	p	*η*^2^_*p*_
Vision condition	1.92	34.50	12.04	<.001	.401
Distance	1.59	28.58	72.00	<.001	.800
Vision condition *x* distance	4.56	82.09	4.25	.002	.191

A follow-up analysis using a Wilcoxon test with Bonferroni correction (see descriptive statistics see S1 Table) revealed no significant differences between the vision conditions at the real distances of 2.0 m. For the real distances of 3.0 m significant difference between the I-RCHB and the two manipulated vision conditions (F-RCH and F-B) are revealed. At a distance of 4.0 m there are significant differences between the incomplete (I-RCHB) and the three full (F-RCHB, F-RCH, and F-B) vision conditions.

#### Variable error (VE)

**Fig 17:**
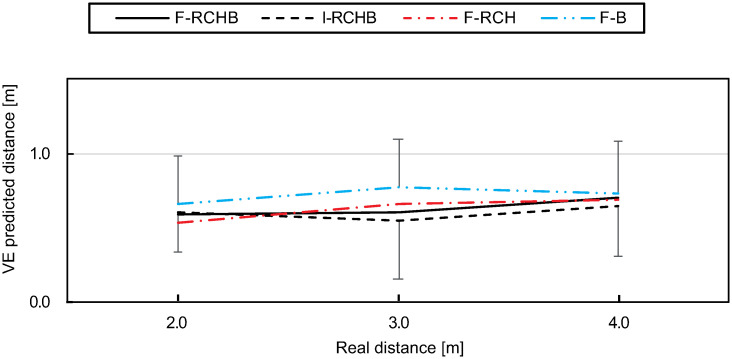
Variable error. Mean and standard deviation of the variable error of the predicted putting distance under the I-RCHB, F-RCHB, F-RCH, and F-B conditions.

The variable error under the F-RCHB condition remains approximately constant from short (0.59 m) to medium (0.60 m) distances and increases to 0.70 m for long distances (see Fig 17 (discriptive statistics see S1 Table). As the only condition, I-RCHB shows a decrease of the variable error from small (0.60 m) to medium (0.55 m) distances with an increase to large (0.65 m) distances. Under condition F-B, the variable error is higher compared to all other conditions for all distances. The variable error increases from short (0.67 m) to medium (0.77 m) distances and decreases for long (0.73 m) distances. An increase from small (0.53 m) to medium (0.67 m) distances of the variable error can also be observed under the F-RCH, the increase continues to large (0.70 m) distances.

The two-factor ANOVA with repeated measures (3 distances: 2.0, 3.0, and 4.0 m; 4 vision conditions: I-RCHB, F-RCHB, F-RCH, and F-B) revealed no significant main effects of vision condition and distance or interaction effect(see Table 17).

**Table 17:** Results of the two-factor ANOVA with repeated measures (3 distances; 4 vision conditions) for the variable error of predicted putt length. Corrected by Greenhouse-Geisser *ϵ*.

Factor	df1	df2	F	p
Vision condition	2.54	45.74	1.35	.271
Distance	2.0	35.93	1.75	.188
Vision condition *x* distance	3.14	56.53	0.38	.779

#### Absolute error (AE)

**Fig 18:**
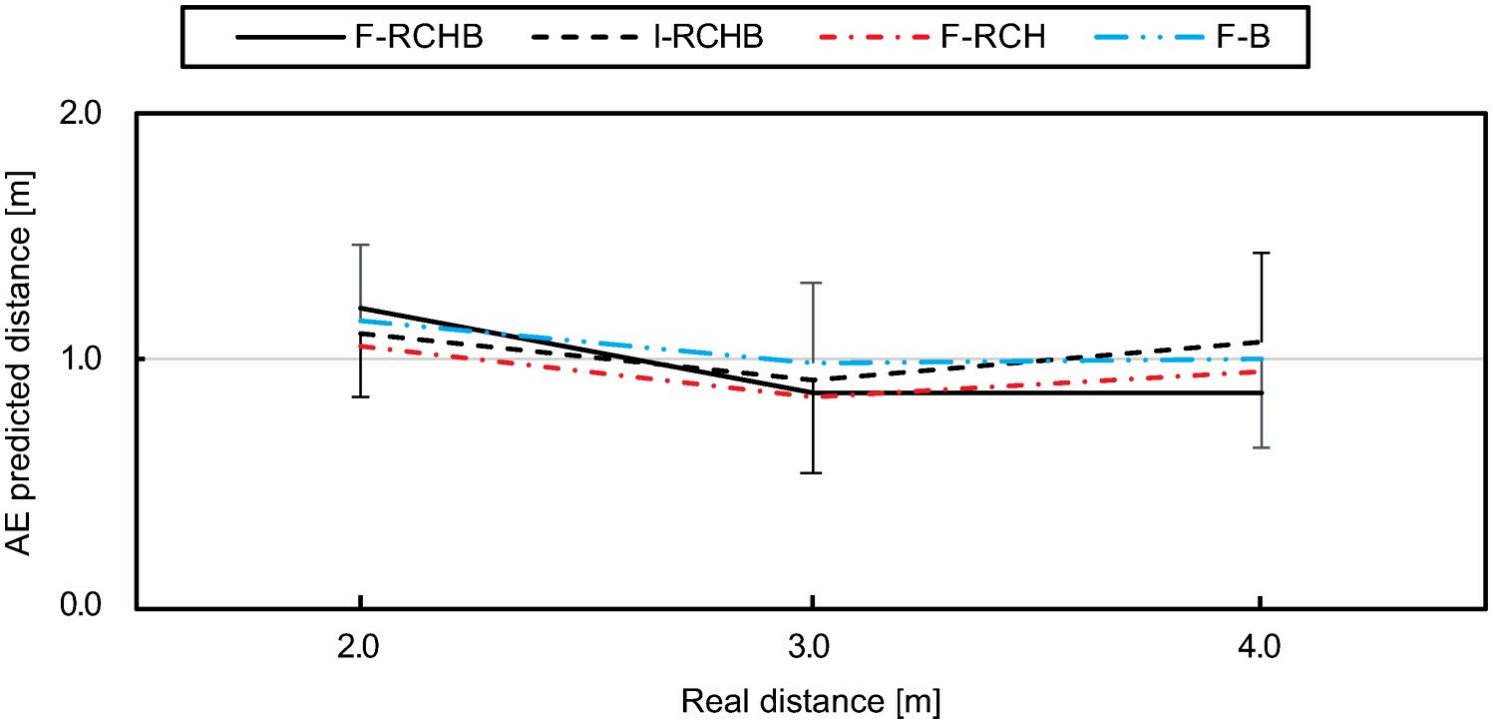
Absolute error. Mean and standard deviation of the absolute error of the predicted putting distance under the F-RCHB, I-RCHB, F-RCH, and F-B conditions.

Fig 18 (descriptive statistics see S1 Table) show a decrease of the absolute error from small to medium distances under all conditions. While under the I-RCHB and F-RCH conditions an increase to large distances follows, the absolute error under the F-RCHB and F-B conditions remains approximately constant.

The two-factor ANOVA with repeated measures (3 distances: 2.0, 3.0, and 4.0 m; 4 vision conditions: F-RCHB, I-RCHB, F-RCH, and F-B) revealed no significant main or interaction effects (see Table 18).

**Table 18:** Results of the two-factor ANOVA with repeated measures (3 distances; 4 vision conditions) for absolute error of the predicted putt length. Corrected by Greenhouse-Geisser *ϵ*.

Factor	df1	df2	F	p
Vision condition	2.36	42.39	0.620	.568
Distance	1.42	25.48	2.77	.097
Vision condition *x* distance	3.66	65.95	0.71	.574

#### Confidence of prediction

**Fig 19:**
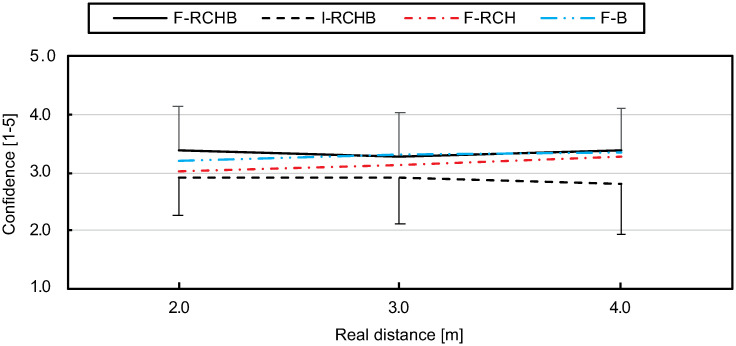
Confidence of prediction. Mean and standard deviation of the confidence of prediction depending on the real putting distance under the I-RCHB, F-RCHB, F-RCH, and F-B conditions.

Compared to the other three conditions, the confidence of the prediction under the I-RCHB condition is lowest over all distances (see Fig 19, descriptive statistics see S1 Table). For small (2.92) and medium (2.92) distances the confidence remains constant and decreases for longer (2.81) distances. The values under the F-RCH condition are higher than the values under I-RCHB and lower than the values under the other two conditions (F-RCHB and F-B) over all distances. The confidence of prediction slightly increases with increasing distance and approaches the values of the F-RCHB and F-B conditions for large distances. Under the F-B condition the confidence of prediction increases with increasing putting distance. The F-RCHB condition shows the highest confidence of prediction for small and large distances, for medium distances the value (3.27) is slightly below the F-B condition (3.3). The confidence of prediction decreases from small to medium distances and increases again from medium to long distances.

The two-factor ANOVA with repeated measures (3 distances: 2.0, 3.0, and 4.0 m; 4 vision conditions: F-RCHB, I-RCHB, F-RCH, and F-B) revealed a significant main effect of vision condition (see Table 19).

**Table 19:** Results of the two-factor ANOVA with repeated measures (3 distances; 4 vision conditions) for the confidence of prediction. Corrected by Greenhouse-Geisser *ϵ*.

Factor	df1	df2	F	p	*η*^2^_*p*_
Vision condition	2.06	36.97	10.87	<.001	.376
Distance	1.45	26.15	1.06	.340	.056
Vision condition *x* distance	3.64	65.55	1.44	.234	.074

A follow-up analysis using a Wilcoxon test with Bonferroni correction (descriptive statistics see S1 Table) revealed significant differences between the F-RCHB and I-RCHB conditions over all distances and between I-RCHB and F-B at the distances of 3.0 and 4.0 m.

#### Response time

**Fig 20:**
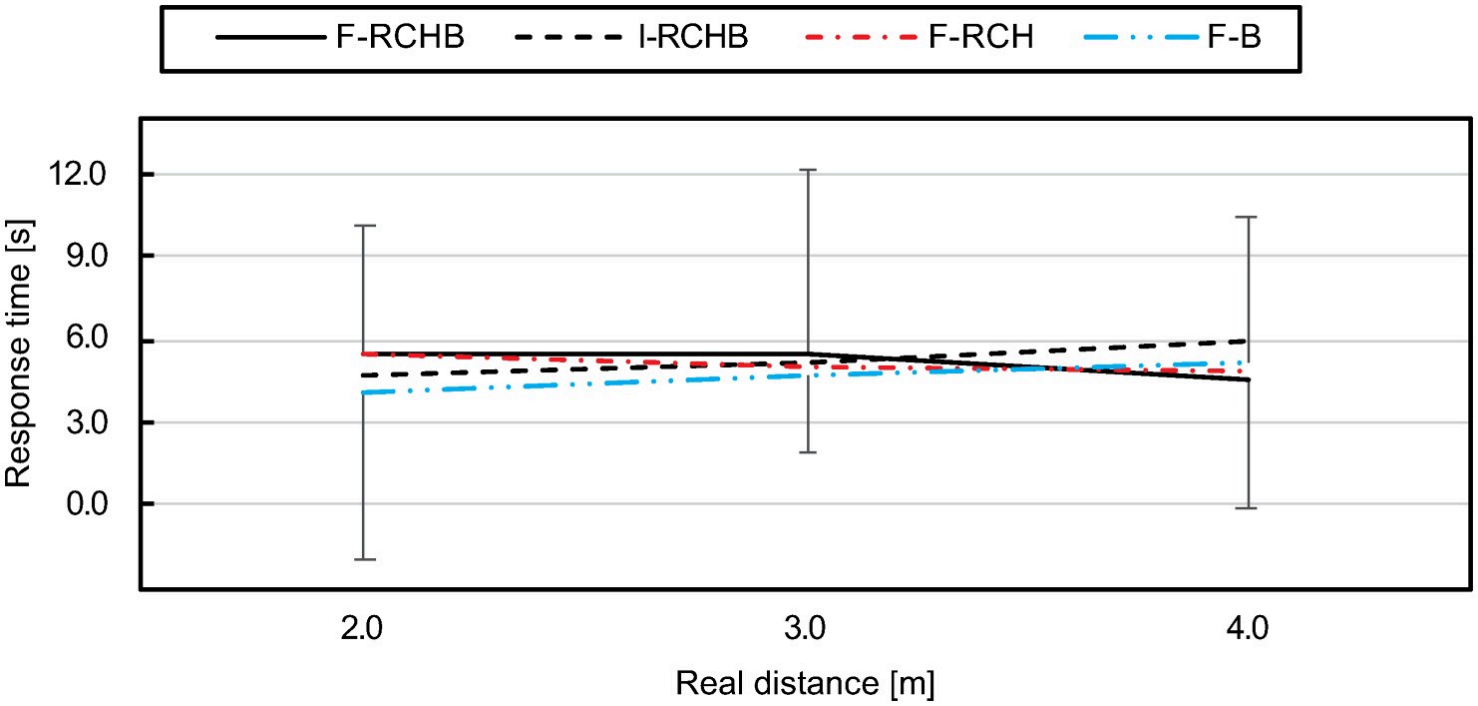
Response time. Mean and standard deviation of the response time depending on the real putting distance under the I-RCHB, F-RCHB, F-RCH, and F-B condition.

Fig 20 (descriptive statistics see S1 Table) show the means and standard deviations of the response time of all participants for the three real putting distances under the four experimental conditions. The response times under the I-RCHB and F-B conditions increase with increasing distance, the response time of F-B is on average 0.65 s below the response time of I-RCHB over all distances. Under F-RCHB, the response time for short and medium distances is higher than under the other conditions, but lowest for long distances. The response time is constant for short and medium distances and decreases from medium to long distances. The response time under the F-RCH condition decreases with increasing putting distance from 5.43 s (2.0 m) to 4.84 s (4.0 m).

The two-factor ANOVA with repeated measures (3 distances: 2.0, 3.0 and 4.0 m; 4 vision conditions: F-RCHB, I-RCHB, F-RCH, and F-B) revealed no significant main or interaction effects (see Table 20).

**Table 20:** Results of the two-factor ANOVA with repeated measures (3 distances; 4 vision conditions) for the response time. Corrected by Greenhouse-Geisser *ϵ*.

Factor	df1	df2	F	p
Vision condition	2.40	43.73	1.02	.388
Distance	1.63	29.39	.183	.790
Vision condition *x* distance	3.88	69.84	1.18	.327

### Discussion of experiment 2

The results show that participants were able to predict the putting distance of a robot putt under the three vision condition with visible follow-through phase. As in experiment 1, all three putting distances were overestimated by the participants, a linear course of the predicted putting distances is shown with increasing real putting distance. This systematic overestimation supports the predictions derived from the FLE. However, contrary to hypothesis 1, prediction error (CE) again does not increase, but rather decreases with increasing putting distance.

In contrast to the three vision conditions with visible follow-through phase, the participants could not predict the putting distance under the incomplete vision condition. As in experiment 1,the predicted values seem to be randomly chosen values that show a tendency to the centre of the prediction scale. The confidence data support the prediction results.

The visibility of the follow-through-phase was found to have a decisive influence on the quality of the prediction of the putting distance. This leads to the conclusion that the additional stimuli available under the three vision conditions with follow-through phase have a high relevance for the prediction of putting movements. It turns out that the visibility of a single element (club or ball) in the swing phase is sufficient for a correct prediction of the putting distance. The combination of visibel elements doesn’t seem to improve the quality of the prediction.

Whereas hypothesis 2 (different prediction depending on vision condition) was confirmed, there was no evidence for hypothesis 1 (increasing error with increasing putting distance) and hypothesis 3 (different prediction depending on vision condition—full view vs. restricted view). Ex post calculations of power revealed that again experiment 2 was over-powered (1.0) regarding hypothesis 2 (Protocol of power analysis see S2 File). With regard to hypothesis 3, a power of 0.79 was calculated (Protocol of power analysis see S2 File).

## General discussion

We performed two experiments to examine the effect of different viewing conditions on accuracy and precision of estimated/predicted putting distance. Comparing the results (distance, CE, AE, VE, confidence, and response time) both studies show comparable values for the respective variables.

First, experiment 1 and experiment 2 reveal a significant over-estimation of putting distance in the full vision conditions. This apparently supports expectations derived from the FLE. However, in contrast to hypothesis 1, prediction error (CE) did not increase, but rather decreased with increasing putting distance. An important difference to the FLE experiments is that under full vision at least one object was still visible after impact. Therefore, this additional information may have destroyed the FLE. In the incomplete condition we also found no increase of prediction error. However, the time of the downswing (0.46 to 0.58 s) may have been too short to allow for valid perception, indicated by CE and confidence. In experiment 1 the CE values show a linear development and a sign change from short distances (1.11 m at a distance of 2.5 m) to long distances (-0.95 m at a distance of 4.0 m). This represents the tendency of the participants to choose distances in the middle of the distance scale at 3.00 m, regardless of the actual distance of the video sequence. At all distances, the confidence in the incomplete condition (mean = 2.63) is below the confidence in the full condition (mean = 3.22). Overall, hypothesis 1 was not supported by the data of either experiment.

Furthermore, experiment 1 reveals significant differences between the predicted putting distance in the full vision (F-RCHB) and the incomplete vision (I-RCHB) condition (see Table 21). While in the full vision condition the predicted putting distance increases with increasing real distance, in the incomplete motion condition the prediction is nearly constant (2.99±0.09 m) around the mean value of the estimation scale (0.00 to 6.00 m) of 3.00 m (see Fig 9). In the full motion condition a systematic overestimation of the putt performance by an average of 0.89±0.18m (mean and standard deviation of the CE) occurs which does not increase with distance (as was expected in hypothesis 1). In the incomplete motion condition, however, the CE shows a change in sign due to the constant prediction around the mean value of the estimation scale and thus a lower mean value of 0.24±0.09 m. Experiment 1 revealed that a valid prediction of the putting distance in the incomplete motion condition (temporal occlusion) is not possible and instead a tendency towards the middle of the estimation scale occurs. This assumption is supported by results of the VE and AE. In both cases there are no significant differences between the two conditions. The prediction of the putting distance shows a constant variability under both conditions. This supports the assumption that a prediction of the distance under the incomplete condition is not possible and that the participants tend to be in the middle of the evaluation scale. The comparable level of the AE can also be explained by the constant overestimation of the putting distance in the full vision condition and the tendency towards the center of the distance scale (at 3.00 m) in the incomplete condition. The confidence of the prediction of the putting distance also shows significant differences between the two conditions depending on the real putting distance (see Fig 13). The prediction of the putting distance in the incomplete motion condition is rated less confident than in the complete condition (mean difference: 0.59 over all distances). The evaluation of the confidence of the prediction supports hypothesis 2 that in the incomplete motion condition a prediction of the putting distance is not possible. Significant differences between the two conditions are also apparent in the response time. Over all distances, the response time in the incomplete motion condition is on average 0.93 s higher than in the complete motion condition, again supporting hypothesis 2. The results of experiment 1 for the prediction of the putting distance, the constant error, the variable error, the absolute error and the confidence of the prediction could be replicated in experiment 2. Therefore, hypothesis 2 was confirmed. Comparable courses of the prediction of the putting distance (see Fig 15), the CE of the prediction (see Fig 16) and the confidence of the prediction (see Fig 19) were found. However, hypothesis 2 could not be replicated regarding response time. While in experiment 1 the response time in the full motion condition is always shorter than in the incomplete motion condition, in experiment 2 it is only consistently shorter in the F-B compared to the I-RCHB condition. The results of the two experiments, which are consistent with regard to the quality and the confidence of prediction, confirm the assumption that the prediction of the putting distance by the human observer is more accurate, more confident and—with some limits—faster when the full motion is shown (hypothesis 2). The follow-through phase of the putt movement has an important influence on the prediction of putt performance. However, hypothesis 1 (increasing errors with increasing putting distance) could not be confirmed in either studies.

**Table 21:** Overview of significant differences in experiment 1 and 2.

Parameter	Experiment 1			Experiment 2		
	Vision condition	Distance	Vision condition *x* distance	Vision condition	Distance	Vision condition *x* distance
Estimated distance	X	X	X	X	X	X
Constant error	X	X	X	X	X	X
Variable error						
Absolut error						
Confidence	X			X		
Response time	X					

The differences in accuracy expected in Experiment 2 in predicting the putting distance between the condition with full vision (F-RCHB) and/or restricted visions, invisible ball (F-RCH) or club and robot (F-B) cannot be confirmed (hypothesis 3). Significant differences can only be demonstrated between the three full conditions and the incomplete condition (I-RCHB). Furthermore, the vision of the ball is not mandatory since the F-RCH condition showed a tendency towards higher accuracy (CE and AE). On the other hand, the invisibility of club, club head and robot seems to be compensated by the vision of the ball. However, the F-RCH condition showed a tendency to be more accurate (estimated distance and CE) than the other full conditions and—with the exception of 4 m distance—the F-B condition resulted in faster response times compared to the other full conditions.

Ex post power analysis regarding hypothesis 2 reveal that both experiments are “overpowered” for the factors predicted distance (power: experiment 1 = 1.0; experiment 2 = 0.999), CE (power: experiment 1 = 1.0; experiment 2 = 0.999) and confidence (power: experiment 1 = 1.0; experiment 2 = 0.999), see S1 and S2 Files. Therefore, the assumed overestimation of the effect size according to the “winner’s curse phenomenon” [40] from experiment 1 was not confirmed in the results of experiment 2. Following Zhang and Hughes [41], a subgroup analysis was carried out. The populations of both studies were divided into two subgroups (group 1: without previous experience; group 2 = with previous experience) based on the mentioned previous experience in golf, field field hockey and similar sports. In experiment 1, 10 participants reported that they had experience in golf or similar sports, 10 participants reported that they had no previous experience. In experiment 2, 8 participants reported that they had experience in golf or similar sports, 11 participants reported that they had no previous experience. The two-factor ANOVAs with repeated measures were calculated with regard to hypothesis 2 for both experiments. Table 22 provides an overview of significant differences in the two studies.

**Table 22:** Overview of *η*^2^_*p*_, significant, and power of the subgroup analyses in experiment 1 and 2.

Subgroup	Parameter	experiment 1		experiment 2	
		Vision condition	Vision condition *x* distance	Vision condition	Vision condition *x* distance
No golf experience	Predicted distance	*η*^2^_*p*_ = .605 (sig.)	*η*^2^_*p*_ = 0.703 (sig.)	*η*^2^_*p*_ = .99 (sig.)	*η*^2^_*p*_ = .99 (sig.)
		power = 1.0	power = 1.0	power = 1.0	power = 1.0
	Constant error	*η*^2^_*p*_ = .605 (sig.)	*η*^2^_*p*_ = .703 (sig.)	*η*^2^_*p*_ = .639 (sig.)	*η*^2^_*p*_ = .639 (sig.)
		power = 1.0	power = 1.0	power = 1.0	power = 1.0
	Confidence	*η*^2^_*p*_ = .804 (sig.)	*η*^2^_*p*_ = .129	*η*^2^_*p*_ = .987 (sig.)	*η*^2^_*p*_ = .403
		power = 1.0	power = .722	power = 1.0	power = .999
Golf experience	Predicted distance	*η*^2^_*p*_ = .686 (sig.)	*η*^2^_*p*_ = 0.681 (sig.)	*η*^2^_*p*_ = .626 (sig.)	*η*^2^_*p*_ = .415
		power = 1.0	power = 1.0	power = .999	power = .927
	Constant error	*η*^2^_*p*_ = .686 (sig.)	*η*^2^_*p*_ = .681 (sig.)	*η*^2^_*p*_ = .415 (sig.)	*η*^2^_*p*_ = .99
		power = 1.0	power = 1.0	power = .999	power = .927
	Confidence	*η*^2^_*p*_ = .573 (sig.)	*η*^2^_*p*_ = .102	*η*^2^_*p*_ = .9 (sig.)	*η*^2^_*p*_ = .206
		power = 1.0	power = .537	power = 1.0	power = .557

The two-factor ANOVA with repeated measures (6 distances: 1.5, 2.0, 2.5, 3.0, 3.5, 240 and 4.0 m; 2 vision conditions: full and incomplete vision) for group 1 in experiment 1 revealed significant main effects of vision condition and distance and no significant interaction effect for the predicted distance and CE. For group 2, significant main effects of vision condition and distance and significant interaction effects were revealed for the predicted distance and the CE. Both groups show significant effects of the vision condition for the confidence (for details see S2 Table). The two-factor ANOVA with repeated measures (3 distances: 2.0, 3.0 and 4.0 m; 4 vision conditions: F-RCHB, I-RCHB, F-RCH, and F-B) for group 1 revealed significant main effects of vision condition and distance and significant interaction effect for the variables predicted distance and CE. For group 2 significant main effects of vision condition and distance and no significant interaction effects were revealed for the predicted distance and the CE. Both groups show significant effects of the vision condition for the confidence (for details see S2 Table). Again, the calculated power analyses regarding the two subgroups revealed consistent overpowerment (with three exceptions: experiment 1—group 1 and 2 regarding interaction of vision condition and distance for confidence; experiment 2—group 2 regarding interaction of vision condition and distance for confidence).

## Conclusion

With regard to the possible spatio-temporal information during the putt motion, stimuli in the downswing phase (t_downswing_ and V_C-impact_) do not seem to be sufficient information for the prediction of putt performance. To test the assumption that the upswing phase has a preparatory function for the perception of the follow-through phase, e.g. eye movement, an isolated study on the influence of the downswing phase on the quality of the prediction of putt performance must be conducted. In the follow-through phase in particular, the duration of the phase seems to be an important indication of information for the putt length. Further combinations of spatio-temporal information, e.g. the ball velocity after the impact (V_B-impact_), the movement of club and ball and X (ball_x follow-through_ and club_x follow-through_) and Y (ball_y follow through_ and club_y follow-through_) direction, as well as the radial distance between club head and ball (rd) do not seem to have a decisive influence on the quality of the prediction. The mentioned spatio-temporal information probably provides redundant information for the human observer.

Based on the results of the two presented experiments, future studies should investigate the influence of different spatio-temporal information in the follow-through phase on the quality of the prediction of the putting distance. The first step is the clarification which elements of the robot putting motion, e.g. ball or club, the human observers pay attention to. The use of an eye-tracking system to record the direction of gaze during the presentation of the individual video sequences represents a feasible approach. In addition, the spatio-temporal information of the follow-through phase should be further differentiated, e.g. various combinations of non-visible robot, club, club head and ball. The ball and the individual elements of the robot-club system, e.g. robot-arm, club, and club head, often represent comparable spatio-temporal information, e.g. club head and robot arm move at the same angular velocity. It is possible that this redundant information has a disruptive influence on the evaluation of the putting performance.

The influence of prior experience in different sports, computer games and golf itself on the prediction of putting performance has to be further investigated. For this purpose, the data already collected provide a basis and should be extended by a group of golf experts, e.g. golf instructors.

Another important extension of the experiments is to test further performance-related parameters to be estimated, e.g., velocity of club and ball at impact, since the distance estimation is outcome-related. This would closer resemble the human-robot interaction where humans have to estimate further performance-related features of robot motion. It is also important to distinguish between absolute judgment and relative judgments. In particular the relative judgments, e.g. slower vs. faster, shorter vs. longer and others are of great importance in a dyadic movement learning process. In the dyad movement learning behavior of human-human dyads, relative judgments and descriptions are often used as feedback, e.g. swing the club more slowly. The further investigation of the transferability of this feedback strategy to human-robot dyads represents an approach to optimize the movement learning process between humans and robots.

## Supporting information

S1 Video 2.00 m robot putt in F-RCHB. Exemplary video sequence of a robot putt with a putting distance of 2.00 m in the full vision condition with visible robot, club, club head, and ball (F-RCHB) in the follow-through phase.
(MP4)

S2 Video 2.00 m robot putt in I-RCHB. Exemplary video sequence of a robot putt with a putting distance of 2.00 m in the incomplete vision condition with visible robot, club, club head, and ball (I-RCHB) in the follow-through phase.
(MP4)

S3 Video 2.00 m robot putt in F-RCH. Exemplary video sequence of a robot putt with a putting distance of 2.00 m in the full vision condition with visible robot, club, and club head. (F-RCH) in the follow-through phase.
(MP4)

S4 Video 2.00 m robot putt in F-B. Exemplary video sequence of a robot putt with a putting distance of 2.00 m in the full vision condition with visible ball (F-B) in the follow-through phase.
(MP4)

S1 Dataset Dataset experiment 1. data set of experiment 1 with the recorded (video sequence number, predicted distance, confidence, and response time) and calculated (constant error, variable error, and absolute error) values.
(XLSX)

S2 Dataset Dataset experiment 2. data set of experiment 2 with the recorded (video sequence number, predicted distance, confidence, and response time) and calculated (constant error, variable error, and absolute error) values.
(XLSX)

S1 File Power calculation 1. G*Power calculation protocol for experiment 1.
(PDF)

S2 File Power calculation 2. G*Power calculation protocol for experiment 2.
(PDF)

S1 Table Descriptive statistics. Descriptive statistics and and results of the follow-up analyzes for experiment 1 and experiment 2.
(PDF)

S2 Table Detailed results of the subgroup analysis. Results of the two-factor ANOVA with repeated measures (6 distances; 2 vision conditions) for the predicted distance, the CE and the confidence for the two subgroups in experiment 1 and of the two-factor ANOVA with repeated measures (3 distances; 4 vision conditions) for the predicted distance, the CE and the confidence for the two subgroups in experiment 2. Corrected by Greenhouse-Geisser *ϵ*.
(PDF)
