# Supplementary material for: Perception and prediction of the putting distance of robot putting movements under different visual/viewing conditions
Source: PLoS One. 2021 Apr 23;16(4):e0249518. doi: 10.1371/journal.pone.0249518 (PMC8064581; doi:10.1371/journal.pone.0249518)
Supplement: S1 File — G*Power calculation protocol for experiment 1. (PDF) [file pone.0249518.s007.pdf]

Power calculation for Study 1

Post hoc: Compute achieved power - hypothesis 2 - predicted distance

F tests - ANOVA: Repeated measures, within factors

**Analysis:**	Post hoc: Compute achieved power		
**Input:**	Effect size f	=	1.3748358
	α err prob	=	0.05
	Total sample size	=	20
	Number of groups	=	2
	Number of measurements	=	6
	Corr among rep measures	=	0.672
	Nonsphericity correction ε	=	1
**Output:**	Noncentrality parameter λ	=	691.5269
	Critical F	=	2.3156892
	Numerator df	=	5.0000000
	Denominator df	=	90.0000000
	Power (1-β err prob)	=	1.0000000

Post hoc: Compute achieved power - hypothesis 2 - constant error

F tests - ANOVA: Repeated measures, within factors

**Analysis:**	Post hoc: Compute achieved power		
**Input:**	Effect size f	=	1.3748358
	α err prob	=	0.05
	Total sample size	=	20
	Number of groups	=	2
	Number of measurements	=	6
	Corr among rep measures	=	0.672
	Nonsphericity correction ε	=	1
**Output:**	Noncentrality parameter λ	=	691.5269
	Critical F	=	2.3156892
	Numerator df	=	5.0000000
	Denominator df	=	90.0000000
	Power (1-β err prob)	=	1.0000000

Post hoc: Compute achieved power - hypothesis 2 - confidence

F tests - ANOVA: Repeated measures, within factors

**Analysis:**	Post hoc: Compute achieved power		
**Input:**	Effect size f	=	1.4746536
	α err prob	=	0.05
	Total sample size	=	20
	Number of groups	=	2
	Number of measurements	=	6
	Corr among rep measures	=	0.546
	Nonsphericity correction ε	=	1
**Output:**	Noncentrality parameter λ	=	574.785
	Critical F	=	2.3156892
	Numerator df	=	5.0000000
	Denominator df	=	90.0000000
	Power (1-β err prob)	=	1.0000000

Post hoc: Compute achieved power - hypothesis 2 - response time

F tests - ANOVA: Repeated measures, within factors

**Analysis:**	Post hoc: Compute achieved power		
**Input:**	Effect size f	=	1.6236883
	α err prob	=	0.05
	Total sample size	=	20
	Number of groups	=	2
	Number of measurements	=	6
	Corr among rep measures	=	0.231
	Nonsphericity correction ε	=	1
**Output:**	Noncentrality parameter λ	=	411.3962
	Critical F	=	2.3156892
	Numerator df	=	5.0000000
	Denominator df	=	90.0000000
	Power (1-β err prob)	=	1.0000000
