# Supplementary material for: Perception and prediction of the putting distance of robot putting movements under different visual/viewing conditions
Source: PLoS One. 2021 Apr 23;16(4):e0249518. doi: 10.1371/journal.pone.0249518 (PMC8064581; doi:10.1371/journal.pone.0249518)
Supplement: S2 File — G*Power calculation protocol for experiment 2. (PDF) [file pone.0249518.s008.pdf]

Power calculation for Study 2

A priori: Compute required sample size - hypothesis 2

F tests - ANOVA: Repeated measures, within factors

**Analysis:**	A priori: Compute required sample size		
**Input:**	Effect size f	=	1.3748358
	α err prob	=	0.05
	Power (1-β err prob)	=	0.80
	Number of groups	=	2
	Number of measurements	=	3
	Corr among rep measures	=	0.672
	Nonsphericity correction ε	=	1
**Output:**	Noncentrality parameter λ	=	69.1526882
	Critical F	=	6.9442719
	Numerator df	=	2.0000000
	Denominator df	=	4.0000000
	Total sample size	=	4
	Actual power	=	0.9975382

A priori: Compute required sample size - hypothesis 3

F tests - ANOVA: Repeated measures, within factors

**Analysis:**	A priori: Compute required sample size		
**Input:**	Effect size f	=	0.5
	α err prob	=	0.05
	Power (1-β err prob)	=	0.80
	Number of groups	=	4
	Number of measurements	=	3
	Corr among rep measures	=	0.5
	Nonsphericity correction ε	=	1
**Output:**	Noncentrality parameter λ	=	18.0000000
	Critical F	=	3.6337235
	Numerator df	=	2.0000000
	Denominator df	=	16.0000000
	Total sample size	=	12
	Actual power	=	0.9408513

Post hoc: Compute achieved power - hypothesis 2 - predicted distance

F tests - ANOVA: Repeated measures, within factors

**Analysis:**	Post hoc: Compute achieved power		
**Input:**	Effect size f	=	0.8181987
	α err prob	=	0.05
	Total sample size	=	19
	Number of groups	=	4
	Number of measurements	=	3
	Corr among rep measures	=	0.250
	Nonsphericity correction ε	=	0.639
**Output:**	Noncentrality parameter λ	=	32.5111267
	Critical F	=	4.0467918
	Numerator df	=	1.2780000
	Denominator df	=	19.1700000

	Power (1-β err prob)	=	0.9994625

Post hoc: Compute achieved power - hypothesis 2 - constant error

F tests - ANOVA: Repeated measures, within factors

**Analysis:**	Post hoc: Compute achieved power		
**Input:**	Effect size f	=	0.8181987
	α err prob	=	0.05
	Total sample size	=	19
	Number of groups	=	4
	Number of measurements	=	3
	Corr among rep measures	=	0.250
	Nonsphericity correction ε	=	0.639
**Output:**	Noncentrality parameter λ	=	32.5111267
	Critical F	=	4.0467918
	Numerator df	=	1.2780000
	Denominator df	=	19.1700000
	Power (1-β err prob)	=	0.9994625

Post hoc: Compute achieved power - hypothesis 2 - confidence

F tests - ANOVA: Repeated measures, within factors

**Analysis:**	Post hoc: Compute achieved power		
**Input:**	Effect size f	=	0.7762500
	α err prob	=	0.05
	Total sample size	=	19
	Number of groups	=	4
	Number of measurements	=	3
	Corr among rep measures	=	0.438
	Nonsphericity correction ε	=	0.685
**Output:**	Noncentrality parameter λ	=	41.8631919
	Critical F	=	3.9193832
	Numerator df	=	1.3700000
	Denominator df	=	20.5500000
	Power (1-β err prob)	=	0.9999625

Post hoc: Compute achieved power - hypothesis 2 - response time

F tests - ANOVA: Repeated measures, within factors

**Analysis:**	Post hoc: Compute achieved power		
**Input:**	Effect size f	=	0.2389193
	α err prob	=	0.05
	Total sample size	=	19
	Number of groups	=	4
	Number of measurements	=	3
	Corr among rep measures	=	0.087
	Nonsphericity correction ε	=	0.810
**Output:**	Noncentrality parameter λ	=	2.8866329
	Critical F	=	3.6341113
	Numerator df	=	1.6200000
	Denominator df	=	24.3000000
	Power (1-β err prob)	=	0.3056498

Post hoc: Compute achieved power - hypothesis 3 - predicted distance

F tests - ANOVA: Repeated measures, within factors

**Analysis:**	Post hoc: Compute achieved power		
**Input:**	Effect size f	=	0.4233737
	α err prob	=	0.05
	Total sample size	=	19
	Number of groups	=	3
	Number of measurements	=	3
	Corr among rep measures	=	0.250
	Nonsphericity correction ε	=	0.702
**Output:**	Noncentrality parameter λ	=	9.5630947
	Critical F	=	3.8434118
	Numerator df	=	1.4040000
	Denominator df	=	22.4640000
	Power (1-β err prob)	=	0.7951375

Post hoc: Compute achieved power - hypothesis 3 - constant error

F tests - ANOVA: Repeated measures, within factors

**Analysis:**	Post hoc: Compute achieved power		
**Input:**	Effect size f	=	0.4233737
	α err prob	=	0.05
	Total sample size	=	19
	Number of groups	=	3
	Number of measurements	=	3
	Corr among rep measures	=	0.250
	Nonsphericity correction ε	=	0.702
**Output:**	Noncentrality parameter λ	=	9.5630947
	Critical F	=	3.8434118
	Numerator df	=	1.4040000
	Denominator df	=	22.4640000
	Power (1-β err prob)	=	0.7951375

Post hoc: Compute achieved power - hypothesis 3 - confidence

F tests - ANOVA: Repeated measures, within factors

**Analysis:**	Post hoc: Compute achieved power		
**Input:**	Effect size f	=	0.4701077
	α err prob	=	0.05
	Total sample size	=	19
	Number of groups	=	3
	Number of measurements	=	3
	Corr among rep measures	=	0.438
	Nonsphericity correction ε	=	0.859
**Output:**	Noncentrality parameter λ	=	19.2542423
	Critical F	=	3.5156504
	Numerator df	=	1.7180000
	Denominator df	=	27.4880000
	Power (1-β err prob)	=	0.9746109

Post hoc: Compute achieved power - hypothesis 3 - response time

F tests - ANOVA: Repeated measures, within factors

**Analysis:**	Post hoc: Compute achieved power		
**Input:**	Effect size f	=	0.2526456
	α err prob	=	0.05
	Total sample size	=	19
	Number of groups	=	3
	Number of measurements	=	3
	Corr among rep measures	=	0.087
	Nonsphericity correction ε	=	0.888
**Output:**	Noncentrality parameter λ	=	3.5386737
	Critical F	=	3.4655441
	Numerator df	=	1.7760000
	Denominator df	=	28.4160000
	Power (1-β err prob)	=	0.3565220
