# Supplementary material for: Perception and prediction of the putting distance of robot putting movements under different visual/viewing conditions
Source: PLoS One. 2021 Apr 23;16(4):e0249518. doi: 10.1371/journal.pone.0249518 (PMC8064581; doi:10.1371/journal.pone.0249518)
Supplement: S1 Table — Descriptive statistics and and results of the follow-up analyzes for experiment 1 and experiment 2. (PDF) [file pone.0249518.s009.pdf]

# Descriptive statistics

**Table 1. Predicted distance under full (F-RCHB) and incomplete (I-RCHB) vision condition (Mean±SD).**

| | Predicted distance [m] | | | | | |
|---|---|---|---|---|---|---|
| **Condition** | **1.5 m** | **2.0 m** | **2.5 m** | **3.0 m** | **3.5 m** | **4.0 m** |
| I-RCHB | 2.61±0.95 | 2.88±1.11 | 3.15±1.06 | 3.15±1.21 | 3.07±1.06 | 3.05±0.99 |
| F-RCHB | 2.47±1.05 | 2.89±0.94 | 3.66±0.99 | 3.83±1.11 | 4.36±1.02 | 4.62±1.00 |

**Table 2. Follow-up analyses (Wilcoxon test with Bonferroni correction) of the interaction of putting distance and vision condition for predicted putting distance.**

| | **1.5 m** | **2.0 m** | **2.5 m** | **3.0 m** | **3.5 m** | **4.0 m** |
|---|---|---|---|---|---|---|
| N | 20 | 20 | 20 | 20 | 20 | 20 |
| Z | -1.755 | -.485 | -3.099 | -3.192 | -3.920 | -3.920 |
| 2p | .079 | .627 | .002* | .001* | <.001* | <.001* |

* Significant after Bonferroni correction. Level of significance $p < .008\overline{3}$.

**Table 3. Constant error of predicted distance under full (F-RCHB) and incomplete (I-RCHB) vision condition (Mean±SD).**

| | Constant error of predicted distance [m] | | | | | |
|---|---|---|---|---|---|---|
| **Condition** | **1.5 m** | **2.0 m** | **2.5 m** | **3.0 m** | **3.5 m** | **4.0 m** |
| I-RCHB | 1.11±0.95 | 0.88±1.11 | 0.65±1.06 | 0.15±1.21 | -0.43±1.06 | -0.95±0.99 |
| F-RCHB | 0.97±1.05 | 0.89±0.94 | 1.16±0.99 | 0.83±1.11 | 0.86±1.02 | 0.62±1.00 |

**Table 4. Follow-up analyses (Wilcoxon test with Bonferroni correction) of the interaction of putting distance and vision condition for constant error.**

| | **1.5 m** | **2.0 m** | **2.5 m** | **3.0 m** | **3.5 m** | **4.0 m** |
|---|---|---|---|---|---|---|
| N | 20 | 20 | 20 | 20 | 20 | 20 |
| Z | -1.755 | -.485 | -3.099 | -3.192 | -3.920 | -3.920 |
| 2p | .079 | .627 | .002* | .001* | <.001* | <.001* |

* Significant after Bonferroni correction. Level of significance $p < .008\overline{3}$.

**Table 5. Variable error of predicted distance under full (F-RCHB) and incomplete (I-RCHB) vision condition (Mean±SD).**

| | Variable error predicted distance [m] | | | | | |
|---|---|---|---|---|---|---|
| **Condition** | **1.5 m** | **2.0 m** | **2.5 m** | **3.0 m** | **3.5 m** | **4.0 m** |
| I-RCHB | 0.58±0.32 | 0.64±0.41 | 0.58±0.41 | 0.72±0.41 | 0.64±0.46 | 0.65±0.35 |
| F-RCHB | 0.65±0.52 | 0.63±0.36 | 0.59±0.27 | 0.63±0.34 | 0.57±0.31 | 0.51±0.30 |

**Table 6. Results of the two-factor ANOVA with repeated measures (6 distances; 2 vision conditions) for the variable error of the predicted putt length. Corrected by Greenhouse-Geisser $\epsilon$.**

| **Factor** | **df1** | **df2** | **F** | **p** |
|---|---|---|---|---|
| Vision condition | 1.00 | 19.00 | 1.09 | .310 |
| Distance | 3.56 | 67.70 | .454 | .748 |
| Vision condition $x$ distance | 3.78 | 71.74 | .808 | .581 |

**Table 7. Absolute error of predicted distance under full (F-RCHB) and incomplete (I-RCHB) vision condition (Mean±SD).**

| | Absolute error predicted distance [m] | | | | | |
|---|---|---|---|---|---|---|
| **Condition** | **1.5 m** | **2.0 m** | **2.5 m** | **3.0 m** | **3.5 m** | **4.0 m** |
| I-RCHB | 1.21±0.81 | 1.12±0.87 | 0.99±0.75 | 0.99±0.69 | 0.91±0.69 | 1.14±0.75 |
| F-RCHB | 1.08±0.92 | 1.00±0.82 | 1.27±0.85 | 1.12±0.80 | 1.11±0.73 | 0.99±0.63 |

**Table 8. Results of the two-factor ANOVA with repeated measures (6 distances; 2 vision conditions) for the absolute error of the predicted putt length. Corrected by Greenhouse-Geisser $\epsilon$.**

| **Factor** | **df1** | **df2** | **F** | **p** |
|---|---|---|---|---|
| Vision condition | 1.00 | 19.00 | .174 | .681 |
| Distance | 2.30 | 43.78 | .356 | .732 |
| Vision condition $x$ distance | 2.50 | 47.45 | 2.05 | .129 |

**Table 9. Confidene of prediction under full (F-RCHB) and incomplete (I-RCHB) vision condition (Mean±SD).**

| | Confidence [1-5] | | | | | |
|---|---|---|---|---|---|---|
| **Condition** | **1.5 m** | **2.0 m** | **2.5 m** | **3.0 m** | **3.5 m** | **4.0 m** |
| I-RCHB | 2.70±0.85 | 2.63±0.83 | 2.56±0.84 | 2.66±0.94 | 2.68±0.83 | 2.57±0.80 |
| F-RCHB | 3.19±0.84 | 3.09±0.81 | 3.18±0.76 | 3.24±0.77 | 3.27±0.81 | 3.40±0.72 |
| Difference | 0.49 | 0.46 | 0.62 | 0.58 | 0.60 | 0.85 |

**Table 10. Response time depending on the real putting distance under full (F-RCHB) and incomplete (I-RCHB) vision condition (Mean±SD).**

| | Response time [s] | | | | | |
|---|---|---|---|---|---|---|
| **Condition** | **1.5 m** | **2.0 m** | **2.5 m** | **3.0 m** | **3.5 m** | **4.0 m** |
| I-RCHB | 5.70±5.87 | 4.47±2.97 | 5.61±5.80 | 5.77±4.55 | 4.99±4.25 | 5.33±4.56 |
| F-RCHB | 4.64±4.54 | 4.35±4.33 | 4.17±4.29 | 5.13±5.68 | 4.61±4.21 | 3.38±3.45 |

**Table 11. Predicted distance under the I-RCHB, F-RCHB, F-RHC, and F-B conditions (Mean±SD).**

| Condition | Predicted distance [m] 2.0 m | 3.0 m | 4.0 m |
|---|---|---|---|
| I-RCHB | 2.82±1.12 | 2.93±1.15 | 3.22±1.07 |
| F-RCHB | 3.12±0.97 | 3.55±0.94 | 4.24±1.04 |
| F-RCH | 2.89±0.95 | 3.44±0.98 | 3.94±1.15 |
| F-B | 3.07±0.86 | 3.71±1.01 | 4.34±1.14 |

**Table 12. Follow-up analyses (Wilcoxon test and Bonferroni correction) of the interaction of putting distance and vision condition for predicted putt length at the real putting distance.**

| | | I-RCHB vs. F-RCHB | I-RCHB vs. F-RCH | I-RCHB vs. F-B | F-RCHB vs. F-RCH | F-RCHB vs. F-B | F-HCB vs. F-B |
|---|---|---|---|---|---|---|---|
| 2.0 m | N | 19 | 19 | 19 | 19 | 19 | 19 |
| | Z | 1.650 | -.322 | -1.368 | -1.408 | -.724 | -1.127 |
| | 2p | .099 | .748 | .171 | .159 | .469 | .260 |
| 3.0 m | N | 19 | 19 | 19 | 19 | 19 | 19 |
| | Z | 2.495 | -2.656 | -3.179 | -.483 | -1.569 | -1.288 |
| | 2p | .013 | .008* | .001* | .629 | .117 | .198 |
| 4.0 m | N | 19 | 19 | 19 | 19 | 19 | 19 |
| | Z | 3.622 | -3.300 | -3.421 | -1.569 | -.262 | -1.569 |
| | 2p | <.001* | .001* | .001* | .117 | .794 | .117 |

* Significant after Bonferroni correction. Level of significance $p < .008\overline{3}$.

**Table 13. Constant error of predicted distance under the I-RCHB, F-RCHB, F-RCH, and F-B conditions (Mean±SD).**

| Condition | CE predicted distance [m] 2.0 m | 3.0 m | 4.0 m |
|---|---|---|---|
| I-RCHB | 0.82±1.15 | -0.07±1.15 | -0.78±1.07 |
| F-RCHB | 1.12±0.97 | 0.55±0.94 | 0.24±1.04 |
| F-RCH | 0.89±0.95 | 0.44±0.98 | -0.06±1.04 |
| F-B | 1.07±0.86 | 0.71±1.01 | 0.34±1.14 |

**Table 14. Follow-up analyses (Wilcoxon test and Bonferroni correction) of the interaction of putting distance and vision condition for the constant error of predicted putt length at the real putting distance.**

| | | I-RCHB vs. F-RCHB | I-RCHB vs. F-RCH | I-RCHB vs. F-B | F-RCHB vs. F-RCH | F-RCHB vs. F-B | F-HCB vs. F-B |
|---|---|---|---|---|---|---|---|
| 2.0 m | N | 19 | 19 | 19 | 19 | 19 | 19 |
| | Z | 1.650 | -.322 | -1.368 | -1.408 | -.724 | -1.127 |
| | 2p | .099 | .748 | .171 | .159 | .469 | .260 |
| 3.0 m | N | 19 | 19 | 19 | 19 | 19 | 19 |
| | Z | 2.495 | -2.656 | -3.179 | -.483 | -1.569 | -1.288 |
| | 2p | .013 | .008* | .001* | .629 | .117 | .198 |
| 4.0 m | N | 19 | 19 | 19 | 19 | 19 | 19 |
| | Z | 3.622 | -3.300 | -3.421 | -1.569 | -.262 | -1.569 |
| | 2p | <.001* | .001* | .001* | .117 | .794 | .117 |

* Significant after Bonferroni correction. Level of significance $p < .008\overline{3}$.

**Table 15. Variable error of predicted distance under the I-RCHB, F-RCHB, F-RCH, and F-B conditions (Mean±SD).**

| | VE predicted distance [m] | | |
|---|---|---|---|
| **Condition** | **2.0 m** | **3.0 m** | **4.0 m** |
| I-RCHB | 0.60±0.26 | 0.55±0.38 | 0.65±0.35 |
| F-RCHB | 0.59±0.32 | 0.60±0.36 | 0.70±0.44 |
| F-RCH | 0.53±0.28 | 0.67±0.40 | 0.70±0.30 |
| F-B | 0.67±0.31 | 0.77±0.32 | 0.73±0.36 |

**Table 16. Results of the two-factor ANOVA with repeated measures (3 distances; 4 vision conditions) for the variable error of predicted putt length. Corrected by Greenhouse-Geisser $\epsilon$.**

| **Factor** | **df1** | **df2** | **F** | **p** |
|---|---|---|---|---|
| Vision condition | 2.54 | 45.74 | 1.35 | .271 |
| Distance | 2.0 | 35.93 | 1.75 | .188 |
| Vision condition $x$ distance | 3.14 | 56.53 | 0.38 | .779 |

**Table 17. Absolute error of the predicted distance under the I-RCHB, F-RCHB, F-RCH, and F-B conditions (Mean±SD).**

| | AE predicted distance [m] | | |
|---|---|---|---|
| **Condition** | **2.0 m** | **3.0 m** | **4.0 m** |
| I-RCHB | 1.12±0.82 | 0.92±0.68 | 1.07±0.78 |
| F-RCHB | 1.22±0.83 | 0.88±0.64 | 0.87±0.60 |
| F-RCH | 1.06±0.74 | 0.85±0.65 | 0.95±0.64 |
| F-B | 1.16±0.74 | 0.99±0.74 | 1.01±0.63 |

**Table 18. Results of the two-factor ANOVA with repeated measures (3 distances; 4 vision conditions) for absolute error of the predicted putt length. Corrected by Greenhouse-Geisser $\epsilon$.**

| **Factor** | **df1** | **df2** | **F** | **p** |
|---|---|---|---|---|
| Vision condition | 2.36 | 42.39 | 0.620 | .568 |
| Distance | 1.42 | 25.48 | 2.77 | .097 |
| Vision condition $x$ distance | 3.66 | 65.95 | 0.71 | .574 |

**Table 19. Confidene of prediction under the I-RCHB, F-RCHB, F-RCH, and F-B conditions (Mean±SD).**

| | Confidence [1-5] | | |
|---|---|---|---|
| **Condition** | **2.0 m** | **3.0 m** | **4.0 m** |
| I-RCHB | 2.92±0.67 | 2.92±0.80 | 2.82±0.87 |
| F-RCHB | 3.38±0.75 | 3.28±0.78 | 3.39±0.71 |
| F-RCH | 3.03±0.83 | 3.13±0.78 | 3.29±0.78 |
| F-B | 3.21±0.72 | 3.30±0.80 | 3.33±0.81 |

**Table 20. Response time depending on the real putting distance under the I-RCHB, F-RCHB, F-RCH, and F-B condition (Mean±SD).**

| | Response time [s] | | |
|---|---|---|---|
| **Condition** | **2.0 m** | **3.0 m** | **4.0 m** |
| I-RCHB | 4.73±3.01 | 5.14±3.34 | 5.92±7.11 |
| F-RCHB | 5.45±4.73 | 5.45±6.69 | 4.57±4.59 |
| F-RCH | 5.43±4.71 | 5.02±3.49 | 4.85±5.97 |
| F-B | 4.07±2.10 | 4.62±2.75 | 5.15±5.20 |
