# Supplementary material for: Perception and prediction of the putting distance of robot putting movements under different visual/viewing conditions
Source: PLoS One. 2021 Apr 23;16(4):e0249518. doi: 10.1371/journal.pone.0249518 (PMC8064581; doi:10.1371/journal.pone.0249518)
Supplement: S2 Table — Results of the two-factor ANOVA with repeated measures (6 distances; 2 vision conditions) for the predicted distance, the CE and the confidence for the two subgroups in experiment 1 and of the two-factor ANOVA with repeated measures (3 distances; 4 vision conditions) for the predicted distance, the CE and the confidence for the two subgroups in experiment 2. Corrected by Greenhouse-Geisser ϵ. (PDF) [file pone.0249518.s010.pdf]

Detailed results of the subgroup analysis

Results of the two-factor ANOVA with repeated measures (6 distances x 2 vision conditions) for the predicted putt length for the two subgroups for study 1. Corrected by Greenhouse-Geisser ε.

Group	Factor	df1	df2	F	p	η^2_p
No golf experience	Vision condition	1.000	9.000	13.775	.005	.605
	Distance	2.176	19.582	20.106	< .001	.691
	Vision condition x distance	2.904	26.135	4.095	< .001	.703
Golf experience	Vision condition	1.000	8.000	17.505	.003	.686
	Distance	1.998	15.985	24.987	< .001	.757
	Vision condition x distance	3.006	24.044	17.071	< .001	.681

Results of the two-factor ANOVA with repeated measures (6 distances x 2 vision conditions) for the constant error of predicted putt length for the two subgroups for study 1. Corrected by Greenhouse-Geisser ε.

Group	Factor	df1	df2	F	p	η^2_p
No golf experience	Vision condition	1.000	9.000	13.775	.005	.605
	Distance	2.176	19.582	18.309	< .001	.670
	Vision condition x distance	2.904	26.135	21.349	< .001	.703
Golf experience	Vision condition	1.000	8.000	17.505	.003	.686
	Distance	1.998	15.985	24.548	< .001	.754
	Vision condition x distance	3.006	24.044	17.071	< .001	.681

Results of the two-factor ANOVA with repeated measures (6 distances x 2 vision conditions) for the confidence of prediction for the two subgroups for study 1. Corrected by Greenhouse-Geisser ε.

Group	Factor	df1	df2	F	p	η^2_p
No golf experience	Vision condition	1.000	9.000	36.810	< .001	8.04
	Distance	3.182	28.634	.969	.425	.097
	Vision condition x distance	3.064	27.570	1.328	.286	.129
Golf experience	Vision condition	1.000	9.000	12.100	.007	.573
	Distance	2.545	22.906	.661	.561	.068
	Vision condition x distance	2.580	23.218	1.022	.392	.102

Results of the two-factor ANOVA with repeated measures (3 distances x 4 vision conditions) for the predicted putt length for the two subgroups for study 2. Corrected by Greenhouse-Geisser ε.

Group	Factor	df1	df2	F	p	η^2_p
No golf experience	Vision condition	1.917	34.499	12.041	< .001	.401
	Distance	1.588	28.580	62.148	< .001	.775
	Vision condition x distance	4.560	82.078	4.247	.002	.191
Golf experience	Vision condition	2.061	14.430	3.994	.041	.363
	Distance	1.317	10.510	35.931	< .001	.837
	Vision condition x distance	2.761	19.327	1.986	.153	.221

Results of the two-factor ANOVA with repeated measures (3 distances x 4 vision conditions) for the constant error of predicted putt length for the two subgroups for study 2. Corrected by Greenhouse-Geisser ε.

Group	Factor	df1	df2	F	p	η^2_p
No golf experience	Vision condition	1.917	34.499	12.041	< .001	.401

	Distance	1.588	28.580	71.997	< .001	.800
	Vision condition x distance	4.560	82.078	4.247	.002	.191
Golf experience	Vision condition	2.061	14.430	3.994	.041	.363
	Distance	1.317	9.217	44.394	< .001	.864
	Vision condition x distance	2.761	19.327	1.986	.153	.221

Results of the two-factor ANOVA with repeated measures (3 distances x 4 vision conditions) for the confidence of prediction for the two subgroups for study 2. Corrected by Greenhouse-Geisser ε.

**Group**	**Factor**	**df1**	**df2**	**F**	**p**	η^2_p
No golf experience	Vision condition	2.054	36.971	10.868	< .001	.376
	Distance	1.452	26.149	1.062	.340	.056
	Vision condition x distance	3.642	65.551	1.441	.234	.074
Golf experience	Vision condition	2.424	16.968	6.772	.005	.492
	Distance	1.410	9.870	.831	.424	.106
	Vision condition x distance	3.043	21.298	.860	.478	.109
